# The miR-5694/AF9/Snail Axis Provides Metastatic Advantages and a Therapeutic Target in Basal-like Breast Cancer

**DOI:** 10.1016/j.ymthe.2020.11.022

**Published:** 2020-11-20

**Authors:** Xin Tian, Hua Yu, Dong Li, Guojiang Jin, Shundong Dai, Pengchao Gong, Cuicui Kong, Xiongjun Wang

**Affiliations:** 1 Cancer Research Institute, The First Affiliated Hospital of China Medical University, Shenyang, China; 2 CAS Key Laboratory of Tissue Microenvironment and Tumor, Institute of Health Sciences, Chinese Academy of Sciences, University of Chinese Academy of Sciences, Shanghai 200031, China; 3 International Peace Maternity and Child Health Hospital, School of Medicine, Shanghai Jiao Tong University, The China Welfare Institute, Shanghai 200030, China; 4 Department of Laboratory Medicine, The First Affiliated Hospital of China Medical University, Shenyang, China; 5 Department of Pathology, The First Affiliated Hospital and College of Basic Medical Sciences of China Medical University, Shenyang, China; 6 Precise Genome Engineering Center, School of Life Sciences, Guangzhou University, Guangzhou 510006, China

**Keywords:** epigenetic regulatory factors, AF9, Snail, breast cancer metastasis, miR-5694, BLBC

## Abstract

Epigenetic deregulation, especially mutagenesis or the abnormal expression of epigenetic regulatory factors (ERFs), plays an important role in malignant tumorigenesis. To screen natural inhibitors of breast cancer metastasis, we adopted small interfering RNAs (siRNAs) to transiently knock down 591 ERF-coding genes in luminal breast cancer MCF-7 cells and found that depletion of *AF9* significantly promoted MCF-7 cell invasion and migration. A mouse model of metastasis further confirmed the suppressive role of AF9 in breast cancer metastasis. RNA profiling revealed enrichment of AF9 targets genes in the epithelial-mesenchymal transition (EMT). Mechanistically, tandem mass spectrometry showed that AF9 interacts with Snail, which hampers Snail transcriptional activity in basal-like breast cancer (BLBC) cells. AF9 reconstitutes an activated state on the promoter of Snail, which is a master regulator of EMT, and derepresses genes by recruiting CBP or GCN5. Additionally, microRNA-5694 (miR-5694) targeted and degraded *AF9* messenger RNA (mRNA) in BLBC cells, further enhancing cell invasion and migration. Notably, AF9 and miR-5694 expression in BLBC clinical samples correlated inversely. Hence, miR-5694 mediates downregulation of AF9 and provides metastatic advantages in BLBC. Restoring expression of the metastasis suppressor AF9 is a possible therapeutic strategy against metastatic breast cancer.

## Graphical Abstract

**Figure:**
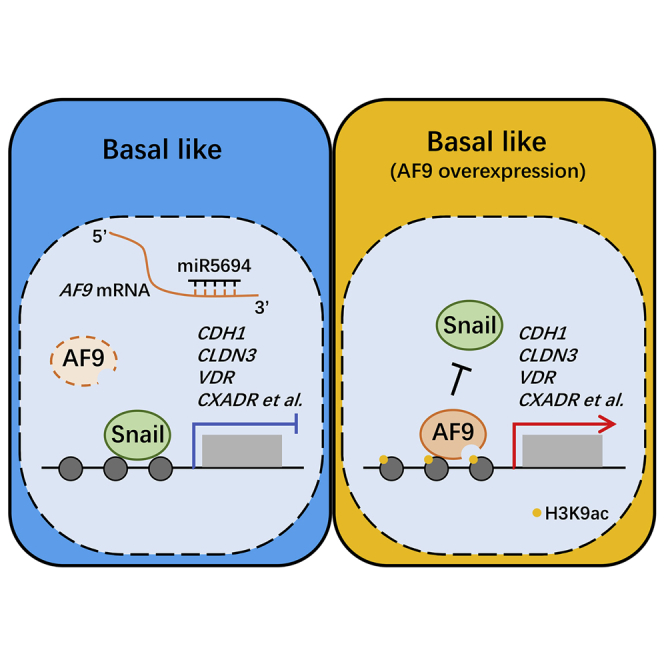

## Teaser

siRNA screening of 591 epigenetic regulatory factors has identified that a histone acetylation reader protein, AF9, represses breast cancer cell migration. miR-5694 downregulates the expression of AF9 and promotes cancer metastasis. This finding may provide a potential target for breast cancer therapy.

## Introduction

Malignant breast cancer can metastasize to lymph nodes and multiple distant organs, such as the lungs, bones, and brain.^1^ Metastasis is the multistep process in which tumor cells detach from primary sites and disseminate to other sites. The prevailing view is that metastatic capacity is a late, acquired event during tumorigenesis and that it is difficult to reverse this transition once metastasis occurs.^2^ Hence, it is particularly important to explore the early regulators initiating cancer metastasis.

Epigenetic deregulation, including gene mutation and silencing, plays an important role in tumorigenesis^3^^,^^4^ and offers potential targets for cancer therapy,^5^ including breast cancer therapy.^6^ Finding natural tumor suppressors to overcome uncontrolled tumor cells, especially to inhibit cancer metastasis, is critical and urgent. Using an *in vitro* small interfering RNA (siRNA) screen covering 591 epigenetic regulatory factor (ERF) coding genes, we initially found that AF9 might be involved in breast cancer cell invasion and migration. The *AF9* gene is also known as mixed-lineage leukemia translocated to chromosome 3 (MLLT3).^7^^,^^8^ The AF9 protein is a subunit of the super elongation complex and associates with the histone H3K79 methyltransferase DOT1L.^9^ It is widely known that MLL-AF9 is the most frequent MLL rearrangement in childhood acute myeloid leukemia (AML).^10^ However, mutations in the AF9 coding region are not associated with leukemia. AF9 has been reported to be tightly associated with neurodevelopmental diseases, such as mental retardation, epilepsy, and ataxia, in human patients.^11^^,^^12^ In addition, depletion of *AF9* in mice phenocopies human neural dysregulation, indicating that AF9 and MLL-AF9 target different downstream genes.^13^^,^^14^ Therefore, AF9 alone could play roles distinct from those of the MLL-AF9 fusion protein. However, whether AF9 contributes to solid tumor development and especially whether AF9 correlates with metastasis remain unknown.

Epithelial-mesenchymal transition (EMT) is a process by which epithelial cells lose their cell polarity and cell-cell adhesion and gain invasive and migratory properties to become mesenchymal cells. Previous studies have shown that the EMT can be regarded as an important symptom of metastasis initiation.^15^ Understanding the causes and consequences of epigenetic dysregulation in the EMT may reveal therapeutic drug targets for metastatic breast cancer.

Snail is a major inducer of the EMT. Snail transcriptional activity correlates positively with cancer progression, which is mainly induced by metastasis, and poor prognosis in various tumors.^16^^,^^17^ Snail represses a wide range of EMT-related genes, such as *CDH1*, *VIM*, *CLDN1*, *CLDN3*, and *VDR*, via epigenetic regulation.^18^^,^^19^ Gene silencing in mammalian cells is usually marked by hypoacetylated or hypermethylated histones at lysine 9 and 27 of histone H3.^20^ Snail interacts with different signaling molecules to regulate epigenetic modifications in histones. Snail requires its N-terminal SNAG domain to interact with several corepressors, including Sin3A, HDAC1/2,^21^ PRC2,^22^ PRMT5,^23^ and LSD1.^24^ Snail transactivation is primarily regulated through the central part of the protein, which contains the most sites of posttranslational modifications.^25^^,^^26^ The C-terminal zinc finger region not only mediates sequence-specific interactions with DNA but is also responsible for the repressor activity of Snail.^27^^,^^28^

In this study, Snail was found to interact with AF9 via its C-terminal domain, and overexpression (OE) of AF9 in basal-like breast cancer (BLBC) cells was shown to impair Snail transcriptional activity. We further observed that AF9 reconstituted an activated state on the promoter of Snail-repressed genes, including *CDH1*, *CLDN3*, *VDR*, and *CXADR*, verifying that AF9 functions as a tumor suppressor, specifically against Snail transactivation, in inhibiting EMT-related genes. As a metastasis suppressor, *AF9* mRNA was degraded by miR-5694 in BLBC. miRNAs interact with their mRNA targets via base-pairing. Most predicted and experimentally characterized miRNA sites are located in the mRNA 3′ UTR, including miR-5694/AF9 mRNA. In TCGA breast cancer database, the expression levels of AF9 mRNA and miR-5694 correlated inversely, further indicating a potential strategy for hampering BLBC metastasis by destroying the miR-5694/AF9 axis.

## Results

### siRNA Screening Reveals that AF9 Plays a Suppressive Role in Breast Cancer Cell Invasion and Migration *In Vitro*

**Figure 1:**
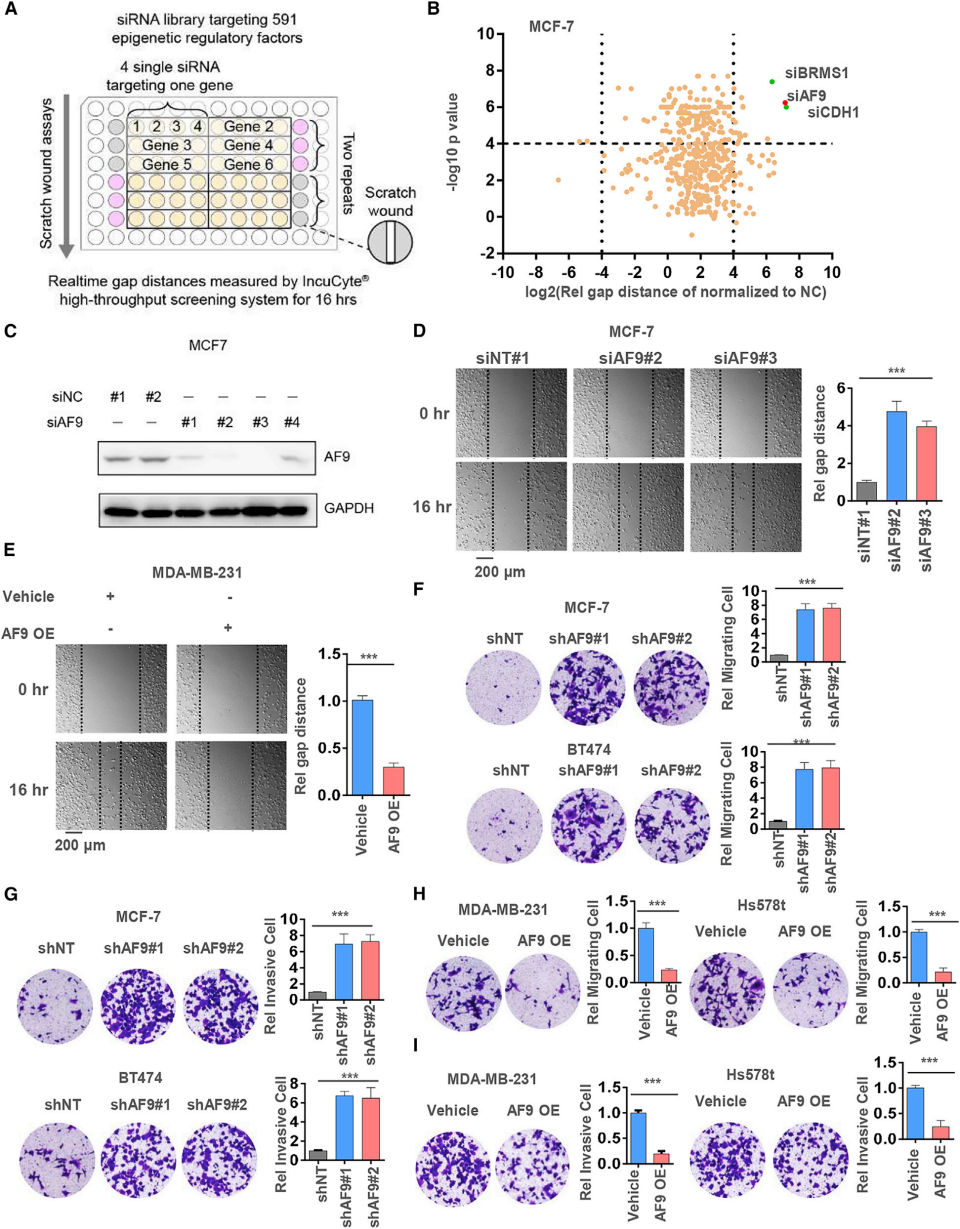
siRNA Screening Reveals that AF9 Plays a Suppressive Role in Breast Cancer Cell Invasion and Migration *In Vitro* (A and B) MCF-7 cells were transfected with individual siRNA targeting 591 epigenetic regulatory factors (ERFs; four siRNA per gene), siRNA targeting *CDH1* or *BRMS1* as the positive control (pink wells) or nontargeting control siRNA (siNC) as the negative control (gray wells). Scratch wound healing assays were performed. Real-time gap distances were measured using an IncuCyte high-throughput screening system for 16 h. Schematic diagram of the screening strategy is presented (A). The data are presented as a volcano plot of two independent experiments (B). The siRNAs with a p value <0.0001 and migration distance (normalized to siNC) <0.8 or >1.2 were considered effective siRNAs that significantly influence tumor cell migration. ERFs targeted by more than two effective siRNAs were selected as candidate ERFs required for tumor cell migration. (C) The protein level of AF9 was examined when siAF9 from 1 to 4 was transfected into MCF-7 cells. siNC from 1 to 2 was used as a nontargeting control. Western blot analysis was performed using an antibody against AF9, and β-actin was also used as an internal control in the following western blot analysis. (D) Representative images of wound healing assays performed in MCF-7 cells transfected with or without siAF9 are shown at the indicated time point (scale bar represents 200 μm). (E) Representative images of wound healing assays performed in MDA-MB-231 cells transfected with or without AF9 overexpression are shown at the indicated time point. Vehicle denotes the empty vector used as a blank control (scale bar represents 200 μm). (F and G) Invasive (F) and migrated (G) Transwell assays of MCF-7-shNT, MCF-7-shAF9#1, #2 cells, T47D-shNT cells, T47D-shAF9#1, and #2 cells. In each group, 4 × 10^4^ cells were plated in serum free medium. After 24 h, the crossed cells were fixed with 70% ethanol, stained with crystal purple, captured by DIC and counted by ImageJ software (scale bar represents 60 μm). (H and I) Invasive (H) and migrated (I) Transwell assays of MDA-MB-231-Vehicle, MDA-MB-231-AF9 OE, Hs578T-Vehicle, and Hs578T-AF9 OE (scale bar represents 60 μm). (F–I) Two-tailed Student’s t test, ∗∗∗p < 0.001. Error bars represent the standard deviation (SD).

In general, BLBC cells, such as MDA-MB-231 and Hs578t cells, display higher metastatic capacity than luminal breast cancer cells, such as MCF-7 and T47D cells.^29^ To identify which ERF contributes to suppressing luminal cancer cell mobility and invasive growth, we adopted siRNAs to knock down 591 genes^30^ encoding ERFs in MCF-7 cells and performed wound healing assays to examine tumor cell migration using an IncuCyte high-throughput screening system (Figure 1A). Among these ERFs, the knockdown of *AF9* significantly enhanced the tumor cells’ wound healing capacity. *CDH1* and *BRMS1* were used as positive controls when their loss drove wound healing (Figure 1B), and *AF9* was depleted in the MCF-7 cells through transient transfection with 4 independent siRNAs in this screening system (Figure 1C). Then, we successfully repeated the wound healing assays using MCF-7 cells with or without *AF9* depletion (Figure 1D). By comparing *AF9* expression in luminal breast cancer cells with high levels, we found quite a low level of the AF9 protein in the BLBC cells (Figure S1A). To further study the function of AF9 in breast cancer, we stably knocked down *AF9* in MCF-7 and T47D cells with two distinct short hairpin RNAs (shRNAs) and induced OE of *AF9* in MDA-MB-231 and Hs578t cells (Figures S1B and S1C). OE of *AF9* inhibited MDA-MB-231 cell invasion according to the wound healing assays (Figure 1E). As wound healing assays involve two phenotypes, i.e., invasion and migration, we separated these two oncological behaviors using invasive and migratory Transwell assays (Figures 1F and 1G). Compared with control cells, OE of *AF9* reduced the cell invasion and migration capacity by approximately 80% in the MDA-MB-231-AF9 OE cells and Hs578T-AF9 OE cells (Figures 1H and 1I). Immunofluorescence (IF) and differential interference contrast (DIC) microscopy showed that morphology changes accompanied AF9 depletion or OE in the MCF-7 or MDA-MB-231 cells, respectively (Figure S1D). Finally, we assessed cell proliferation when *AF9* was depleted in MCF-7 and T47D cells or OE in MDA-MB-231 and Hs578t cells. As shown in Figure S1E, AF9 had no significant effect on breast cancer cell proliferation (Figure S1E). Based on the above data, we preliminarily concluded that AF9 plays a suppressive role in breast cancer invasion and migration but not cell proliferation.

### AF9 Expression Level Determines Acquired Metastatic Capacity in Luminal Breast Cancer Cells and BLBC Cells

**Figure 2:**
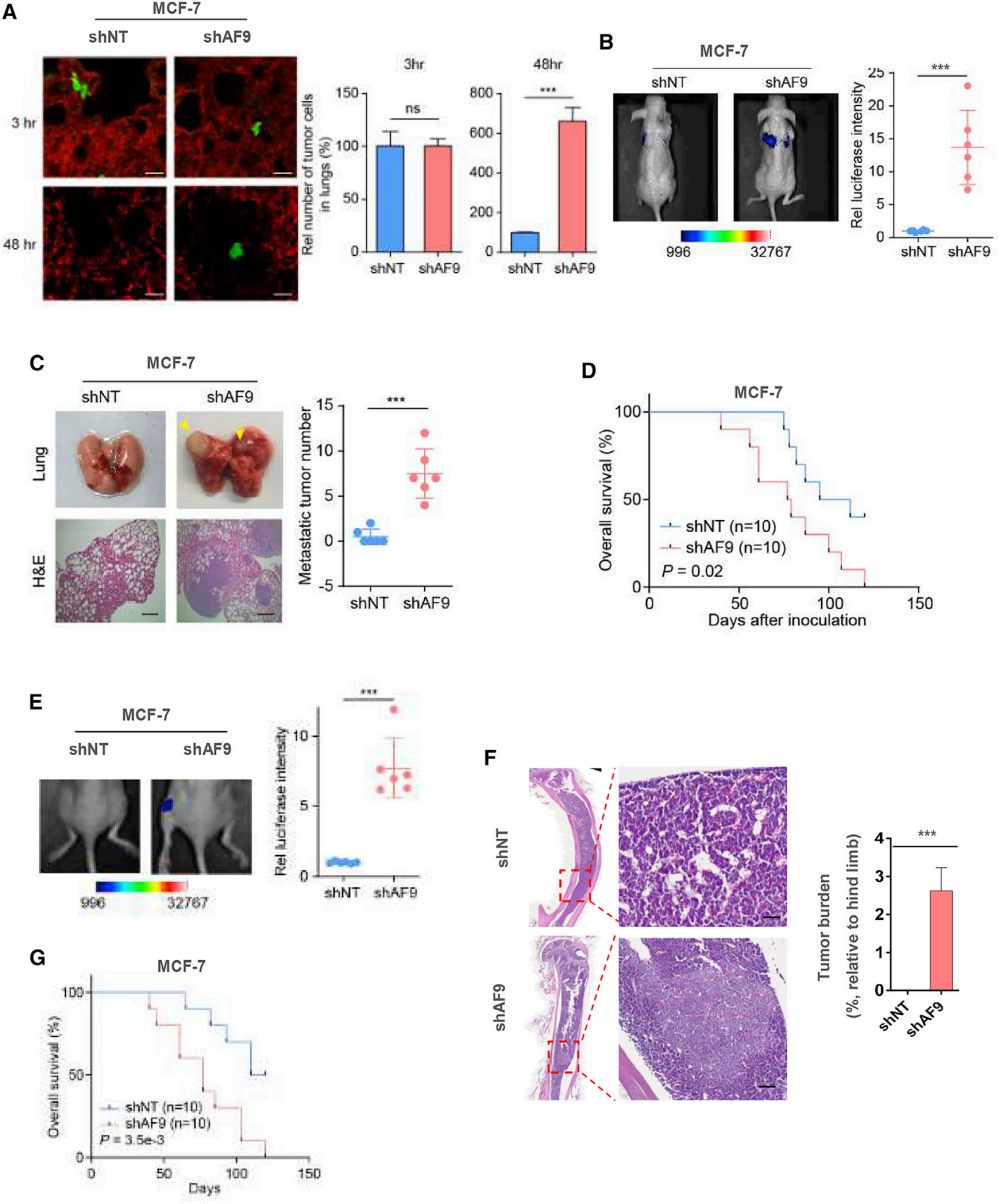
AF9 Expression Level Determines the Acquired Metastatic Capacity of Luminal Breast Cancer and BLBC Cells *In Vivo* (A) Extravasation analysis of MCF-7 cells. MCF-7-shNT cells or MCF-7-shAF9 cells were implanted into randomized athymic nude mice via a tail vein injection (5 mice per group). Then, 3 or 48 h after the inoculation, the mice were sacrificed. Representative images of extravascular tumor cells (green) out of blood vessels (red) are shown (A, left panel). The numbers of extravascular tumor cells were quantified (A, right panel). Scale bar, 20 μm. (B and C) Mouse model of lung metastasis. MCF-7-shNT cells or MCF-7-shAF9 cells were implanted into nude mice via a tail vein injection (6 mice per group). Then, 75 days after the inoculation, bioluminescence imaging of these implanted mice was carried out, and representative images of lung metastasis are presented (B, left panel). The luciferase intensities of the metastatic tumors in the lungs were statistically analyzed (B, right panel). Representative images of lung tissues dissected 90 days after the inoculation and H&E stained metastatic nodules are presented (C, left panel) and were calculated by counting the surface tumors in the lungs (C, right panel). The data represent the mean ± SD of the luciferase intensities in 6 mice. Scale bar, 200 μm. (D) Survival durations. Kaplan-Meier survival analysis of another batch of mice (10 mice per group) implanted with tumor cells as described in Figure 2A. (E and F), Mouse model of *in situ* intraductal-transplantation. Tumors were initiated by injection of 5 × 10^4^ MCF-7 cells into nude mice via intraductal-transplantation. Nude mice in the control group were given 0.1 mL RPMI 1640. The *in situ* tumors were dissected and removed by surgery when reaching 5 mm^2^. After 75 days, the metastatic tumors were first visualized by bioluminescence imaging of luciferase activity. Real time images are presented, and the intensities of the images are shown (E). Then, the tumors were dissected and snap-frozen for the molecular biology analyses. The bone metastatic site was identified by H&E staining, and the relative metastatic area was counted and is presented as a ratio of the whole hind leg bone (F). Scale bar, 400 μm. (G) Survival durations. Kaplan-Meier survival analysis of another batch of mice (10 mice per group) implanted with the following tumor cells as described in Figure 2E: MCF-7-shNT cells or MCF-7-shAF9 cells. (A–C, E, and F) Two-tailed Student’s t test, ∗∗∗p < 0.001. ns, not significant. Error bars represent the standard deviation (SD).

To measure breast cancer cell dissemination *in vivo*, we injected MCF-7-shNT cells and MCF-7-shAF9 cells or MDA-MB-231-Vehicle cells and MDA-MB-231-AF9 OE cells into mice via the tail vein. The mice were sacrificed 3 or 48 h after the inoculation. Representative images of extravascular tumor cells (green) outside of blood vessels (red) are shown in Figures 2A and S2A, left panel. The number of extravascular tumor cells was determined (Figure 2A; Figure S2A, right panel). According to extravasation analysis, depletion of *AF9* improved the luminal breast cancer cell dissemination capacity; in contrast, OE of AF9 impaired BLBC cell dissemination, suggesting that the AF9 expression level determines acquired metastatic capacity *in vivo* independent of the cell type (luminal breast cancer cells or BLBC cells).

To examine the consequence of AF9-mediated breast cancer metastasis, we implanted cells into nude mice via the tail vein (Figure 2B; Figure S2B). At 75 days in the MCF-7 cells or 25 days in the MDA-MB-231 cells after inoculation, bioluminescence imaging of these implanted mice was conducted, and representative images of the metastatic sites are presented in Figures 2B and S2B, left panel. The luciferase intensities of the metastatic tumors in the lungs were statistically analyzed and showed that the MCF-7-shAF9 and MDA-MB-231-Vehicle cells colonized and migrated into the lungs of the nude mice, while the MCF-7-shNT cells did not obviously metastasize, and the MDA-MB-231-AF9 OE cells presented a robust decrease in lung colonization (Figure 2B, right panel). Representative images of H&E-stained metastatic tumor nodules in lung tissue dissected after the inoculation are presented in Figures 2C and S2C. Consistently, the inoculation of the MCF-7-shAF9 cells reduced the survival time of the nude mice, while the inoculation of the MDA-MB-231-AF9 OE cells prolonged the survival time of the nude mice compared to the inoculation of the control group cells (Figure 2D; Figure S2D).

Because the tail vein injection model displays the process of cell migration, extravasation, and colonization *in vivo*, it cannot represent the entire development of *in situ* breast cancer, especially the progression of local invasion to distant organ metastasis. Hence, we injected human breast cancer cells with genetic manipulation into the breast pad of nude mice. Once the tumors reached 5 mm^2^, we removed the tumors via surgery. At 75 days in the MCF-7 cells or 25 days in the MDA-MB-231 cells after surgery, bioluminescence imaging of the implanted mice was conducted, and representative images of the metastatic sites are presented in Figures 2E and S2E, left panel. The luciferase intensities of the metastatic tumors in the hind leg bones, lungs, and even brains were statistically analyzed, and the results showed that the MCF-7-shAF9 and MDA-MB-231-Vehicle cells colonized and migrated into the distant organs of the nude mice, while the MCF-7-shNT cells did not obviously metastasize, and the MDA-MB-231-AF9 OE cells presented a decrease in metastatic colonization of approximately 50% (Figure 2E; Figure S2E, right panel). Representative images of H&E-stained invaded tumor nodules of bone lesions dissected after the inoculation are presented and analyzed in Figures 2F and S2F. Finally, in this *in situ* inoculation model, the overall survival (OS) of the nude mice is similar to that of the mice in the tail vein model (Figure 2G; Figure S2G). Overall, the reliable screening strategy and subsequent *in vitro* and *in vivo* experiments strongly confirm that AF9 functions as a suppressor of breast cancer metastasis.

### AF9 Regulates Expression of EMT-Related Genes

**Figure 3:**
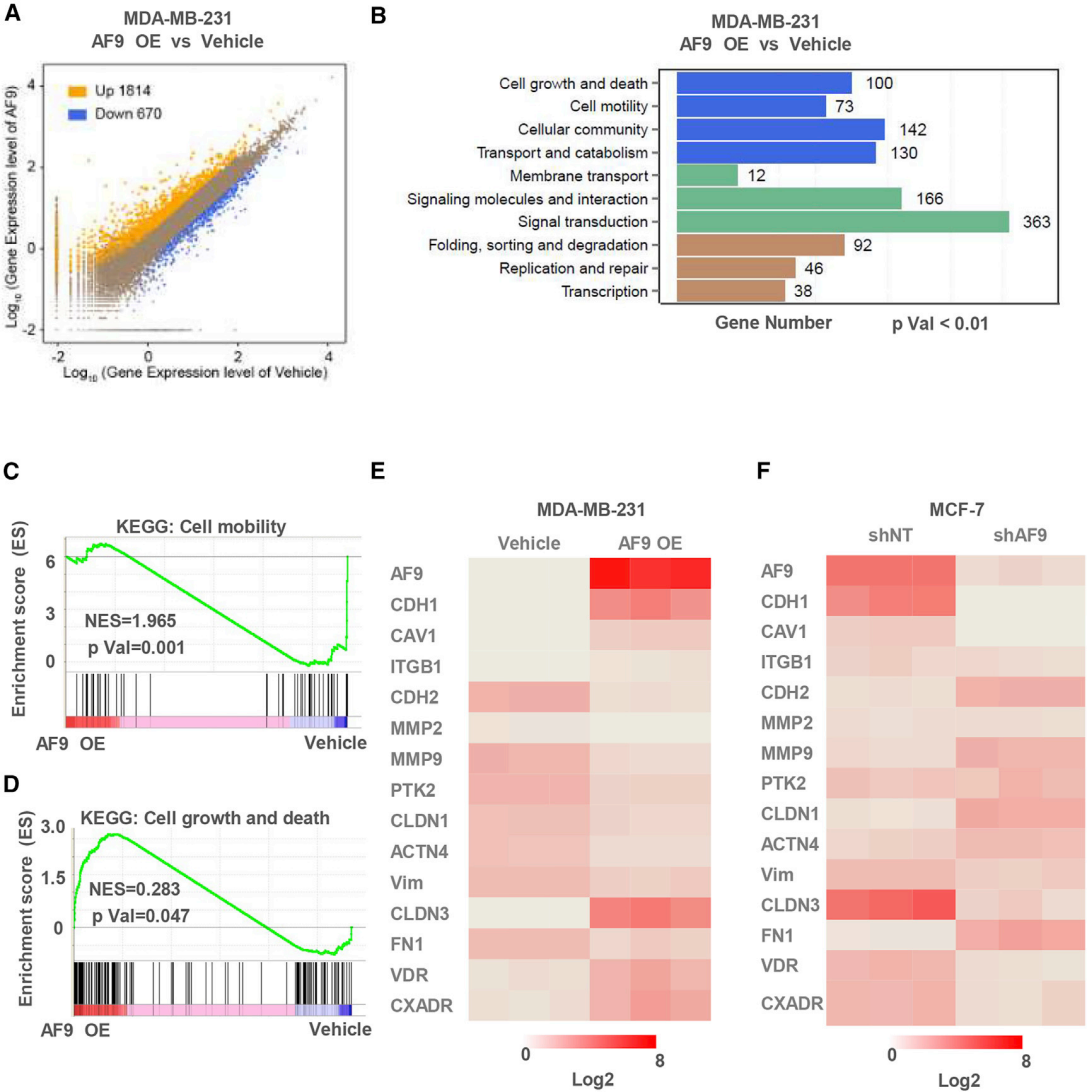
AF9 Regulates the Expression of EMT-Related Genes (A–D) In MDA-MB-231-Vehicle cells and MDA-MB-231-AF9 OE cells, RNA-seq was performed to investigate the AF9-regulated gene profiles. (A) Scatterplot showing that overexpression of AF9 increased the expression of 1,814 genes and decreased the expression of 670 genes. (B) The top 11 pathways (scoring by gene number and p value) using a Kyoto Encyclopedia of Genes and Genomes (KEGG) pathway analysis with a p value cutoff < 0.01, and the gene number is shown in the figure. (C and D) Gene Set Enrichment Analysis (GSEA) of cell mobility or cell growth and death are shown. (E) 14 genes regulating cell mobility were identified by qPCR with a ratio value of the gene expression levels of the AF9 OE group versus the Vehicle group of the MDA-MB-231 cells. The data are representative of at least three independent experiments. (F) 14 genes regulating cell mobility were identified by qRT-PCR with a ratio value of the gene expression levels of MCF-7-shNT cells and MCF-7-shAF9 cells. The data are representative of at least three independent experiments.

To explore the mechanism by which AF9 regulates cell invasion and migration, we applied RNA sequence profiling to expand the AF9-regulated gene profile. By comparing the expression profiles of the MDA-MB-231-Vec cells and MDA-MB-231-AF9 OE cells, we observed that OE of AF9 led to the upregulation of 1,814 genes and downregulation of 670 genes (Figure 3A), which, overall, is consistent with the function of AF9 as a transcription coactivator.^31^ The Kyoto Encyclopedia of Genes and Genomes (KEGG) pathway analysis revealed the top 10 KEGG pathways (Figure 3B). Among the top 10 pathways, cell motility-related genes ranked second. The Gene Set Enrichment Analysis (GSEA) showed that the AF9-regulated genes were significantly involved in cell mobility (Figure 3C) but only slightly affected cell growth and death (Figure 3D), which is consistent with the previous results shown in Figures 1 and S2. In the MDA-MB-231-Vehicle and MDA-MB-231-AF9 OE cells or MCF-7-shNT and MCF-7-shAF9 cells, quantitative reverse transcriptase PCR (qRT-PCR) and western blotting (WB) were performed to confirm the expression of multiple EMT-related genes, including *CDH1*, *CLDN1/3*, *VIM*, and *MMP2/9*^32^ (Figures 3E and 3F; Figures S3A and S3B). Notably, *CDH1* and *CLDN3* were the two most prominent genes among the genes upregulated or downregulated by OE or depletion of *AF9*, indicating that AF9 could positively regulate *CDH1* and *CLDN3* expression (Figures 3E and 3F). The IF analysis showed that the membrane location of CDH1, CDH2, and CDH1 exhibited an enhanced level following *AF9* OE (Figure S3C). To validate the status of the EMT influenced by AF9, we recorded the cellular morphology and analyzed the width to length ratio of the cells (Figure S3D).

### AF9 Interacts with Snail and Hampers Snail Transcriptional Activity

**Figure 4:**
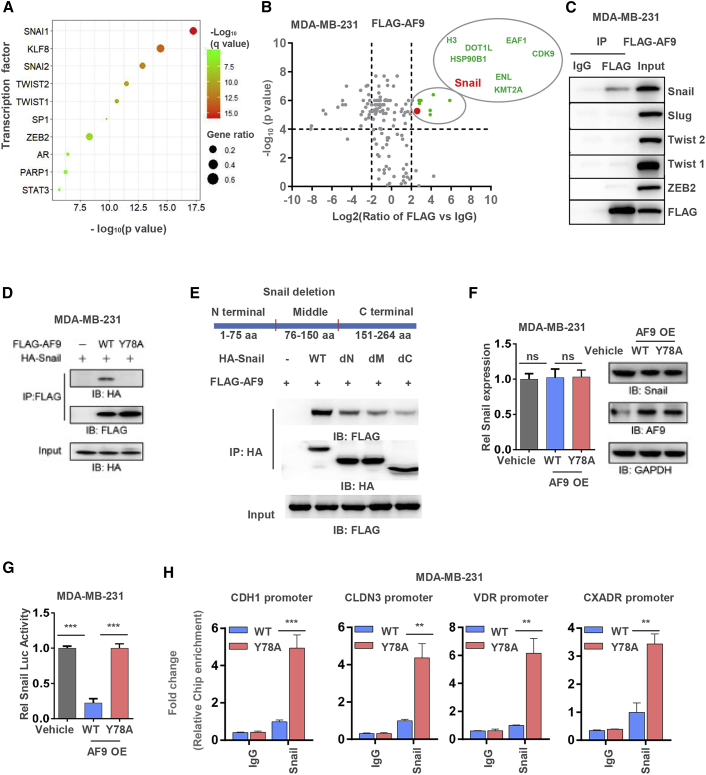
AF9 Interacts with Snail and Hampers Snail Transcriptional Activity (A) Transcription factor (TF) enrichment of AF9 target genes demonstrated that most genes were potentially regulated by SNAI1 transcription factor, with q value less than 0.0001 considered statistically significant. Description of this method is provided in the Materials and Methods section. (B) Mass spectrometry analyses of AF9-associated proteins in MDA-MB-231 cells. The data are presented by a volcano plot. Proteins with a p value <0.0001 and Log_2_ (FLAG-AF9 versus FLAG) >2.0 were regarded as candidate proteins that significantly interact with AF9. The enlarged red point indicates Snail; other significant proteins are marked as green points. All significant proteins are listed in the larger circle. (C) In MDA-MB-231-FLAG-AF9 OE cells, M2 FLAG beads was used to immunoprecipitate the AF9 associated complex. Western blot analysis was performed using the indicated antibodies. (D) HA-Snail and FLAG-AF9 (WT or Y78A) were cotransfected into MDA-MB-231 cells. Antibody against FLAG was used to immune-precipitate the FLAG-AF9 associated complex. Western blot analysis was performed to test the interaction between Snail and AF9 using the indicated antibodies. (E) Separate deletion of three Snail regions (N-terminal, middle domain, and C-terminal), as shown in the upper panel. FLAG-AF9 and HA-Snail (including WT, dN ter, dM, and dC ter) were cotransfected into MDA-MB-231 cells. Antibody against HA was used to immunoprecipitate HA-Snail full length and truncated Snail associated complex. Ter, terminal; M, middle. (F) Snail mRNA expression and protein levels were tested in MDA-MB-231-Vehicle and MDA-MB-231-AF9 OE (WT or Y78A) cells. (G) Snail transcriptional activity was determined in MDA-MB-231-Vehicle and MDA-MB-231-AF9 OE (WT or Y78A) cells by a dual luciferase assay. These cells were cotransfected with the pGL3-*CDH1* promoter (−57 to +149 relative to TSS) and Renilla control plasmid pRL-TK for 48 h. The relative luciferase activities were normalized to those of the cells expressing vehicle and then those of the Renilla control. The data represent the mean ± SD of three independent experiments. (H) ChIP assay was performed in MDA-MB-231-AF9 OE (WT or Y78A) cells with indicated the antibodies. Primers against the promoter regions of *CDH1*, *CLDN3*, *VDR*, and *CXADR* were used to perform quantitative real-time PCR to measure the binding affinity of Snail. The calculation of the relative binding affinities of Snail to the target regions is described in the methods section. (F–H) Two-tailed Student’s t test, ∗∗p < 0.01, ∗∗∗p < 0.001. ns, not significant. Error bars represent the standard deviation (SD).

Certain classical transcriptional factors (TFs), such as Snail, Slug, Twist1/2, and ZEB1/2, are involved in mediating expression of EMT-related genes.^33^ 14 of the AF9-modulated EMT-related genes were predicted to be regulated by 10 TFs, including Snail, Slug, Twist1/2, and ZEB2 (Figure 4A). Furthermore, we performed a mass spectrometry (MS) analysis of the AF9-associated proteins in the MDA-MB-231-FLAG-AF9 OE cells. Among these proteins, eight were found to likely significantly interact with AF9. Notably, these eight proteins are all chromatin-associated proteins. We marked Snail with an enlarged red point and listed the other seven proteins, all of which are likely associated with AF9 (Figure 4B). Coimmunoprecipitation (coIP) was performed to confirm this interaction between AF9 and Snail, Slug, Twist1/2, and ZEB2 in MDA-MB-231-FLAG-AF9 OE cells. Snail, but not the other TFs, was found in the AF9-associated complex (Figure 4C). To test whether the interaction between AF9 and Snail depends on the ability of AF9 to recognize lysine acetylation, we mutated tyrosine (Y) 78 to alanine (A) 78. Y78 is located in the YEATS domain of AF9, and a mutation of Y78 to A78 should abolish the capacity of AF9 to recognize histone lysine acetylation.^31^ As shown in Figure 4D, AF9 bearing the Y78A mutation mostly lost the ability to interact with Snail. To determine the region of Snail that binds AF9, we separately deleted the N-terminal, middle, and C-terminal regions from the whole length of Snail. Reverse coIP was performed, and we observed that the destruction of the integrity of the Snail protein impaired the interaction between Snail and AF9 (Figure 4E). However, both the mRNA and protein expression levels of Snail were not affected by the AF9 OE in the MDA-MB-231 cells and Hs578T cells (Figure 4F; Figure S4A). OE of AF9 dramatically reduced (Vehicle versus AF9 OE = ∼100% versus ∼25%) the TF activity of Snail (Figure 4G; Figure S4B). A chromatin IP (ChIP) assay further revealed that Snail bound the promoter of *CDH1*, *CLDN3*, *VDR*, and *CXADR* and that OE of AF9 heavily disrupted the binding affinity of Snail to these promoters in both MDA-MB-231 cells and Hs578T cells (Figure 4H; Figures S4C and 4D). Thus, we speculated that AF9 disturbed Snail binding the promoters of *CDH1*, *CLDN3*, *VDR*, and *CXADR*, which may explain why AF9 impaired the Snail-mediated suppression of *CDH1*, *CLDN3*, *VDR*, and *CXADR*. Notably, in addition to *CDH1* and *CLDN3*, *VDR*, and *CXADR* have been shown to be important proteins involved in the EMT.^34^^,^^35^

### AF9 Is Required for Reconstituting an Activated State on the Promoter of Snail-Repressed Genes

**Figure 5:**
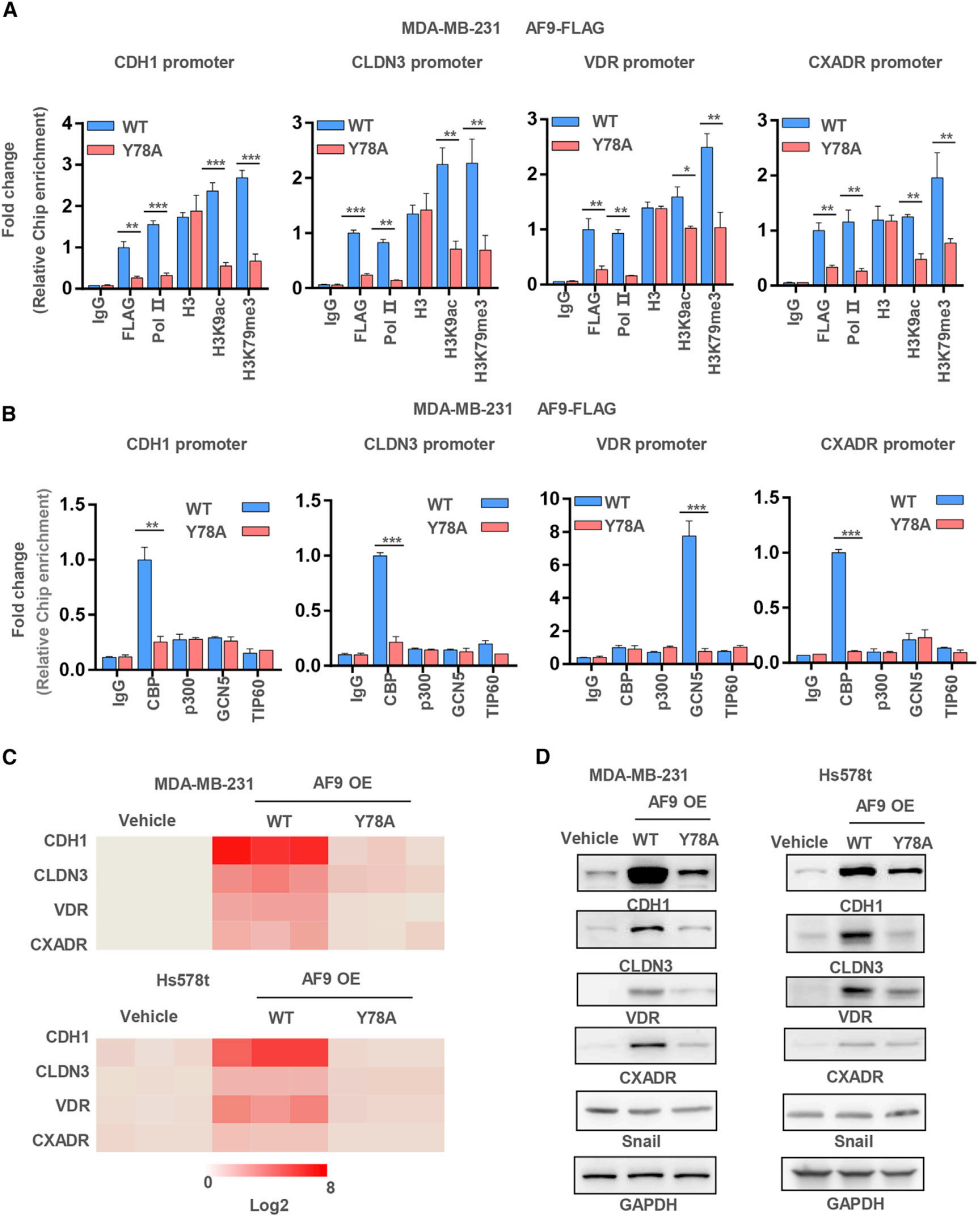
AF9 Is Required for Reconstituting an Activated State on the Promoter of Snail-Repressed Genes (A and B) ChIP assay was performed in MDA-MB-231-FLAG-AF9 OE (WT or Y78A) cells with the indicated antibodies. Primers against the promoter regions of *CDH1*, *CLDN3*, *VDR*, and *CXADR* were used to perform quantitative real-time PCR to measure the binding affinity of activated gene expression markers and histone acetyltransferases to these genes’ promoters. The relative binding affinities were normalized to the WT group with a FLAG antibody (A) or CBP antibody (B). Two-tailed Student’s t test, ∗p < 0.05, ∗∗p < 0.01, ∗∗∗p < 0.001. ns, not significant. Error bars represent the standard deviation (SD). (C and D) mRNA and protein levels of *CDH1*, *CLDN3*, *VDR*, and *CXADR* were tested in MDA-MB-231 and Hs578T cells with the indicated manipulations.

We speculated that AF9-driven *CDH1*, *CLDN3*, *VDR*, and *CXADR* expression could couple with the ability of AF9 as described in a previous report.^36^ However, whether AF9 is required for reconstituting an activated state on the gene promoter is still unknown. To test this hypothesis, we performed a ChIP assay in MDA-MB-231-FLAG-AF9 OE cells using antibodies against FLAG, Pol II, H3, H3K9ac, and H3K79me3. Pol II and H3K9ac were used as activated state markers of gene transcription. As shown in Figure 5A, FLAG, Pol II, H3K9ac, and H3K79me3 bound the *CDH1*, *CLDN3*, *VDR*, and *CXADR* promoters in MDA-MB-231-FLAG-AF9 OE (wild-type [WT]) cells, while these associations were dramatically reduced in the Y78A mutant cells. Because the Y78A mutant lost the ability to recognize H3K9ac, we suspected that the H3K9ac level on the promoters of the *CDH1*, *CLDN3*, *VDR*, and *CXADR* genes was downregulated by a potential feedback loop (Figure 5A). We also restored the expression of AF9, including in the WT and Y78A mutant MCF-7-shAF9 cells (Figure S5A), and further tested the association between AF9, Pol II, H3K9ac, and H3K79me3 and the *CDH1* promoter; the results showed that restored expression of the Y78A mutant AF9 could not establish an activated state on the *CDH1* promoter (Figure S5B).

To discover this potential feedback loop, we sought to determine which of the four important nucleus histone acetyltransferases, i.e., CBP, p300, GCN5, and TIP60, could be recruited to the promoters of the *CDH1*, *CLDN3*, *VDR*, and *CXADR* genes in the MDA-MB-231-FLAG-AF9 OE (including WT and Y78A mutant) cells. As shown in Figure 5B, CBP was recruited to the promoters of the *CDH1*, *CLDN3*, and *CXADR* genes, while GCN5 was recruited to the promoter of the *VDR* gene in the MDA-MB-231-FLAG-AF9 OE (WT) cells. Interestingly, in the Y78A mutant cells, recruitment of histone acetyltransferases to the promoters of the *CDH1*, *CLDN3*, *VDR*, and *CXADR* genes was greatly decreased, suggesting that the YEATS domain of AF9 is required for reconstituting an activated state on the promoters of Snail-repressed genes. Thus, AF9 exerts its ability not only by recognizing H3K9ac but also actively recruiting acetyltransferases to modify histones, which, in turn, establishes an activated state for gene expression (Figure 5B). Consistently, in the MCF-7 cells with restored Y78A mutant expression, AF9 lost the ability to recruit CBP to the *CDH1* promoter (Figure S5C). In addition, we used the positive control target regions of p300 and TIP60 to exclude the possibility of antibodies against p300 and TIP60 unsuitable for ChIP (Figure S5D).

As a result of AF9 reconstituting the activated state on the promoters, the mRNA and protein expression levels of the *CDH1*, *CLDN3*, *VDR*, and *CXADR* genes correlated positively with AF9 expression and its ability to recognize lysine acetylation (Figures 5C and 5D; Figures S5E and S5F).

### miR-5694 Targets and Destabilizes *AF9* mRNA

**Figure 6:**
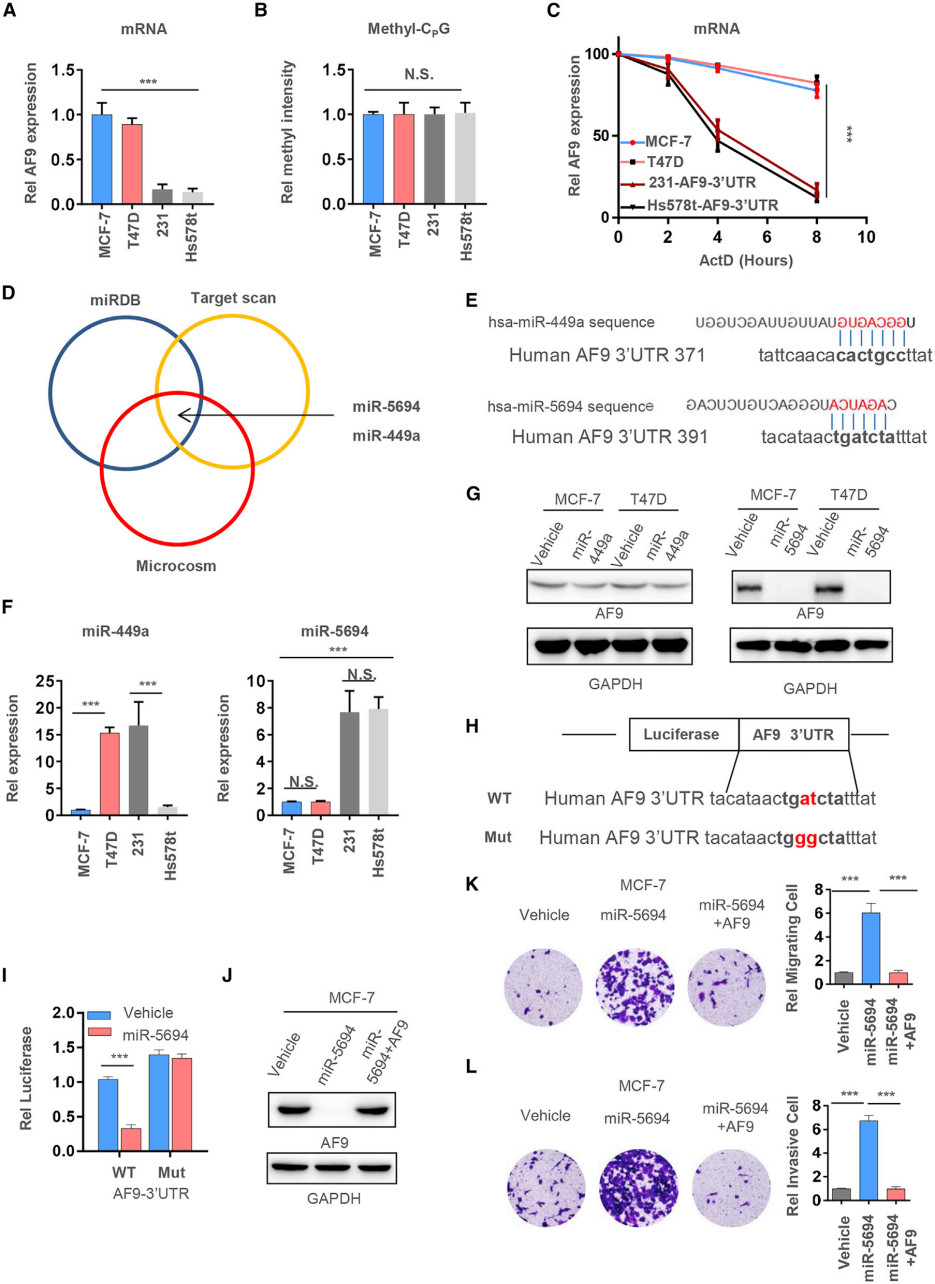
miR-5694 Targets and Destabilizes AF9 mRNA (A) mRNA levels of the AF9 gene were tested in MCF-7, T47D, MDA-MB-231, and Hs578T cells. qRT-PCR was performed using primers specifically against AF9 mRNA. (B) Methylation-specific PCR was used to amplify the PHGDH promoter from MCF-7, T47D, MDA-MB-231, and Hs578T cells and pyrosequencing searched the methylation site in CpG islands. (C) Actinomycin D (named Act D, 1 μM)-treated MCF-7, T47D, MDA-MB-231, and Hs578T cells for the indicated time points and cells were collected for the AF9 mRNA quantification. qRT-PCR was performed using primers specifically against AF9 mRNA. (D) miRDB database (http://mirdb.org/index.html), target scan database (www.targetscan.org), and Microcosm targets (https://omictools.com/microcosm-targets-tool) were used to predict the potential miRNAs regulating AF9 gene expression, and the miRNAs with the top two prediction scores were retained. (E) Schematic diagram of the miRNAs potentially targeting AF9 mRNA 3′ UTR. (F) Expression levels of miR449a and miR5694 in MCF-7, T47D, MDA-MB-231, and Hs578T cells were tested by qPCR using specific primers against miR449a or miR5694, respectively. (G) AF9 proteins were tested by a western blot analysis in MCF-7 and T47D cells with or without the overexpression of miR5694. (H) Schematic diagram of AF9 3′ UTR mutagenesis for abolishing mir-5694 targeting sites. (I) AF9 mRNA was tested in MCF-7 cells containing WT or mutated AF9 mRNA 3′ UTR after the overexpression of miR5694. (J) AF9 proteins were tested by a WB analysis in MCF-7 cells expressing vehicle, miR-5694, or miR-5694+AF9. (K and L) Invasive (K) and migrated (L) Transwell assays were performed using the cells constructed in (J). (A, B, F, I, K, and L) Two-tailed Student’s t test, ∗∗∗p < 0.001. ns, not significant. (C) Two-way analysis of variance (ANOVA), ∗∗∗p < 0.001. Error bars represent the standard deviation (SD).

AF9 suppressed cell invasion and migration in luminal breast cancer cells; however, AF9 was downregulated in the BLBC cells to a certain extent, which explained the higher invasion and migration ability of the BLBC cells. To discover the mechanisms regulating the expression level of AF9, we first tested *AF9* mRNA in two luminal cell lines, i.e., MCF-7 and T47D, and two BLBC cell lines, i.e., MDA-MB-231and Hs578t. As shown in Figure 6A, the *AF9* mRNA level in the luminal cells was approximately five times that in the BLBC cells (Figure 6A). Unexpectedly, there were no differences in the methylation of CpG islands in the *AF9* promoter among these four cell lines (Figure 6B), suggesting that a difference in the stability of posttranscriptional mRNA exists between luminal and BLBC cells. Actinomycin D, which is an inhibitor that blocks transcription by preventing RNA pol elongation, suppresses nascent mRNA generation. As MDA-MB-231 and Hs578t contain low levels of *AF9* mRNA, we reintroduced cytomegalovirus (CMV) promoter-driven expression of the *AF9* coding region and its 3′ UTR. As shown in Figure 6C, when these four cell lines were treated with actinomycin D, we observed an obvious difference in mRNA stability. Generally, RNA binding proteins, such as HuR, bind mRNA 3′ UTRs and stabilize mRNA,^37^ while miRNAs interact with their mRNA targets via base-pairing. Most predicted and experimentally characterized miRNA sites are positioned in the mRNA 3′ UTR. Hence, we predicted that the *AF9* 3′ UTR could be targeted by the following two miRNAs: miR-449a and miR-5694 (Figures 6D and 6E). However, expression of miR-5694 in the BLBC cells was higher than that in the luminal cells, but expression of miR-449a was higher in the T47D and MDA-MB-213 cells and lower in the MCF-7 and Hs578t cells, indicating that the expression level of miR-449a does not correlate with the migration ability of these breast cancer cell lines (Figure 6F). Furthermore, OE of miR-449a did not reduce AF9 expression, while miR-5694 completely degraded *AF9* mRNA in the MCF-7 and T47D cells (Figure 6G). After mutating the 3′ UTR of WT *AF9* to ablate the miR-5694 binding site, the mRNA levels of *AF9* remained similar between the Vehicle and miR-5694 OE groups, and the reintroduction of AF9 with the pCDNA3.1 plasmid into the miR-5694 OE cells restored the *AF9* protein level, further demonstrating that miR-5694 directly targets the 3′ UTR of *AF9* mRNA (Figures 6H–6J). In addition, the improvement in invasion and migration in the MCF-7 cells induced by miR-5694 OE could be diminished by forced expression of AF9, offering a potential strategy against metastasis initiation (Figures 6K and 6L). We also designed an antigomiR to specifically downregulate miR-5694 (designated antigomiR-5694; Figure S6A). As expected, transient transfection with antigomiR-5694 efficiently reduced the level of miR-5694 and recovered the AF9 mRNA level, while the control antigomiR had no effect on miR-5694 or AF9 mRNA (Figures S6B and S6C).

### In Clinical Samples, the *AF9* mRNA Level Correlates Inversely with miR-5694 and Its Reduction Marks a Malignant Prognosis in Breast Cancer Patients

**Figure 7:**
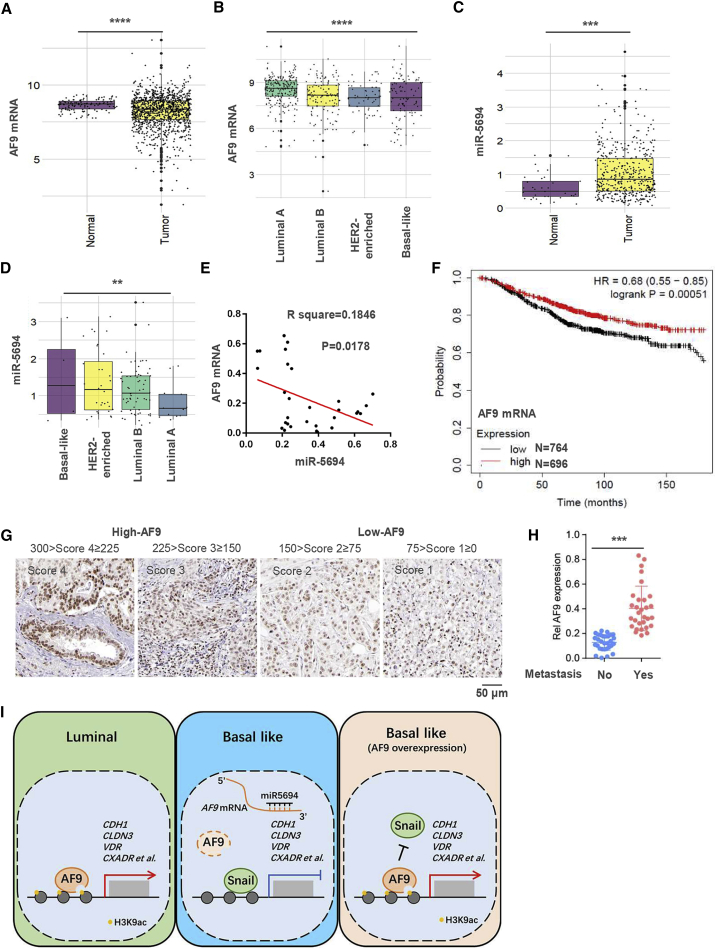
AF9 mRNA Level in Clinical Samples Correlates Inversely with miR-5694 Expression and Serves as a Biomarker of Malignant Breast Cancer (A and B) Boxplots of AF9 and miR-5694 expression in primary breast tumors and adjacent normal tissues in TCGA-BRCA cohort. (C and D) Quantitative analysis of AF9 and miR-5694 expression among four breast cancer subtypes in TCGA-BRCA cohort. Boxplots represent the median and interquartile range (IQR). (E) Correlation between AF9 and miR-5694 expression in clinical BLBC samples. (F) AF9 expression level in tumors prolongs breast cancer patients’ survival time. The data were mined from the Kaplan-Meier plotter database. (G) IHC analysis of breast cancer patients using anti-AF9 antibodies were performed. Images of four representative specimens (scale bar represents 50 μm). We divided the specimens into the following two groups according to the IHC scores: High-AF9 and Low-AF9. (H) Primary tumor tissues were collected from patients with or without metastasis. AF9 mRNA levels were tested in these tissues. (I) Schematic diagram of this study. Generally, we discovered a suppressor of breast cancer metastasis, AF9, which presented extremely low expression in BLBC cells due to miR-5694. In luminal A breast cancer cells or forced expression of AF9 in BLBC cells, AF9 interacts with Snail and hampers Snail transcriptional activity. Then, AF9 reconstitutes an activated state for EMT-related gene expression. (A–D and H) Two-tailed Student’s t test, ∗∗∗p < 0.001. (E) Pearson’s correlation test. Error bars represent the standard deviation (SD).

The clinical role of AF9 in solid tumors has not been elucidated, especially whether AF9 functions as a metastasis suppressor and its association with miR-5694 in clinical breast cancer samples. First, we data mined *AF9* mRNA in normal and tumor tissues from breast cancer patients in TCGA database. As shown in Figure 7A, the *AF9* mRNA level in tumors was significantly lower than that in normal tissue. Breast cancer is approximately divided into certain types based on the origin of its cell type, such as luminal A, luminal B, and BLBC, and the molecules expressed on the cell surface, such as Her2-enriched or triple-negative breast cancer (TNBC). BLBC is commonly known as TNBC because most cases lack expression of estrogen and progesterone receptors and exhibit the OE and/or amplification of *HER2.*^38^ The *AF9* mRNA levels decrease sequentially in luminal A, luminal B, Her2-enriched, and BLBC, indicating that AF9 could function as a potential breast cancer suppressor in the clinic. Using the same batch but a smaller number of samples, we mined miR-5694 expression in normal and tumor tissues, including luminal A, luminal B, Her2-enriched, and BLBC tissues. As shown in Figures 7C and 7D, expression of miR-5694 in the different tissue or tumor types seems to overall correlate inversely with the *AF9* mRNA level (Figures 7C and 7D). We further quantified this reverse correlation with a significant p and R-squared values using samples with detailed clinical information, such as the stage (Figure 7E).

Using the Kaplan-Meier-plotter database, the OS curve of breast cancer patients showed that lower expression of *AF9* mRNA correlated with a shorter survival time of the patients, suggesting poor outcomes and prognosis (Figure 7F). To assess the correlation between *AF9* expression and metastasis, we collected primary tumor tissues from patients with high or low distant organ metastasis. Levels of AF9 protein in tissues were scored into four grades (Figure 7G), and the patients with multiple organ metastasis expressed low AF9 levels (Figure 7H), suggesting that reduced AF9 expression marks malignant progression in breast cancer patients.

## Discussion

Tumor metastasis has been regarded as the most important risk factor for patient survival.^15^^,^^39^ To inhibit or delay the process of metastasis, the discovery of natural suppressors of metastatic breast cancer is a potential strategy. In general, luminal-derived breast cancer is not as malignant as BLBC because BLBC cells have a higher ability to invade and metastasize, causing multiple organ metastasis and placing patients at a high risk.^40^, ^41^, ^42^ The luminal A breast cancer cell lines (MCF-7 and T47D) display low invasion and migration, while the basal-like cell line (MDA-MB-231) presents higher metastasis to bone, lung, and brain in an intraductal-transplantation mouse model.^43^^,^^44^ The transition of ductal carcinoma *in situ* (DCIS) to invasive carcinoma (IC) in breast cancer marks malignant progression and is a high-risk factor. Hence, restraining metastatic breast cancer by tapping potential metastasis suppressors in low invasion and migration cells and verifying their inhibitory effect in highly metastatic BLBC cells represent an effective strategy.

ERFs participate in the most extensive regulation of gene expression and, therefore, are considered very promising clinical drug targets. To identify inhibitory ERFs, we applied siRNAs to target 591 ERF-coding genes in MCF-7 cells and found that AF9 showed the potential to suppress wound healing, suggesting that AF9 may be a suppressor of cell invasion, migration, or both. As a consequence of *AF9* depletion in luminal A breast cancer cells, we observed an increase in cell invasion and migration *in vitro*. Unfortunately, the endogenous *AF9* mRNA level was too low to detect in the following two BLBC cell lines: MDA-MB-231 cells and Hs578T cells. As expected, forced expression of AF9 inhibited both cell invasion and migration.

In this study, we first identified that AF9 is a natural suppressor of breast cancer cell invasion and migration by using siRNAs targeting 591 ERF-coding genes, and a series of *in vitro* experiments confirmed this finding. Second, the following two *in vivo* metastatic models were constructed: extravasation analysis via tail vein injection and an intraductal-transplantation mouse model; these models were used to measure the metastatic capacity of human breast cancer cells with genetic manipulation. Then, we observed that AF9 regulates a cluster of EMT-related genes. Mechanistically, AF9 interacts with Snail and hampers Snail transcriptional activity; in turn, AF9 recruits the histone acetyltransferase CBP or GCN5 to reconstitute an activated state on the promoter of certain EMT-related genes. In addition, we found that miR-5694 targeted and destabilized the AF9 mRNA 3′ UTR in BLBC cells. Finally, low levels of *AF9* expression were found to correlate with poor clinical outcomes and prognosis in breast cancer patients (Figure 7I).

Although metastasis involves multiple steps, invasion and migration are two major steps in metastasis initiation.^45^^,^^46^ To fully evaluate the effect of AF9 on cell invasion and migration ability *in vivo*, we carefully applied two mouse models in this study. First, tumor cells were injected into the tail vein to avoid the first step of metastasis, i.e., local invasion, and this model directly measured cell migration, extravasation, and even colonization. Second, an intraductal-transplantation mouse model was used to comprehensively assess the ability of human breast cancer cell metastasis because the model involves all metastasis steps, including local invasion, migration, extravasation, and colonization. As a luminal A cell line, MCF-7 cells have limited invasion and migration capacities *in vivo*, but when *AF9* was depleted in the MCF-7 cells, we observed a metastatic site in the hind bone. Moreover, compared to injection of MDA-MB-231-Vehicle cells into nude mice, forced expression of AF9 in MDA-MB-231 cells prolonged the survival time of the mice by an average of 8 days. The integrated utilization of the two animal models convinced us that AF9 certainly inhibits metastasis in BLBC cells and that loss of AF9 promotes cancer metastasis. Obviously, it is essential, but difficult, to enhance AF9 expression to antagonize breast cancer progression, especially its metastasis to bones.

Then, we applied RNA sequencing (RNA-seq), bioinformatics web prediction, and liquid chromatography-MS (LC/MS) to uncover the mechanism by which AF9 regulates downstream target genes. First, RNA-seq was used to obtain the expression profile of the Vehicle and AF9 OE groups. Among the differentially expressed genes regulated by AF9, a cohort of EMT-related genes caught our attention. EMT is a profound event that occurs not only in tumor cells but also in embryonic development.^47^^,^^48^ The bioinformatics web software prediction revealed that some well-known TFs, such as Snail, Slug, Twist1/2, ZEB2, and even STAT3, could be involved in mediating these EMT-related genes. LC-MS is another powerful method used for detailed mechanistic evaluations, especially to explain how AF9 increases EMT gene expression. As shown, Snail was found to be the only transcription factor in the AF9-associated complex. Specifically, AF9 interacts with Snail through its C terminus to impair the transcriptional activity of Snail, especially on the promoters of *CDH1*, *CLDN3*, *VDR*, and *CXADR*. Because the C terminus of Snail contains four Zn finger domains,^49^ we suspect that the binding of AF9 to Snail prevents Snail from efficiently targeting its DNA-binding motif.

Another key question is how AF9 can reconstitute an open state on the promoter regions of *CDH1*, *CLDN3*, *VDR*, and *CXADR* after the removal of Snail. Global histone H3 acetylation marks a ready state of gene expression.^50^^,^^51^ AF9 contains a YEATS domain in its N terminus, which recognizes H3 lysine acetylation, especially H3K9ac.^36^ AF9 bearing the Y78A mutation in the YEATS domain is known to lose the ability to bind H3K9ac. As expected, we found that the Y78A mutant lost its association with H3K9ac on the promoters of *CDH1*, *CLDN3*, *VDR*, and *CXADR*. However, the H3K9ac levels on these promoters also decreased, indicating that a negative feedback loop exists between the recognition and maintenance of H3K9ac. Consistently, the recruitment of CBP or GCN5 to these promoters was decreased or not detected in the Y78A mutant cells. The detailed regulatory mechanism between H3K9ac recognition and maintenance is still elusive. Moreover, Li et al.^31^ observed a global decrease in H3 lysine acetylation after the depletion of *AF9.*

In general, tumor suppressors are silenced at the gene expression level or mutated at the protein level in malignant cancer cells; thus, determining how to restore tumor suppression requires in-depth investigations.^52^ In this study, we predicted potential miRNAs targeting the AF9 mRNA 3′ UTR and found that miR-449a and miR-5694 were the most capable candidates with the top two scores. A previous report showed that miR-564 inhibited prostate cancer metastasis and proliferation and identified AF9 as a likely target of miR-564,^53^ indicating that AF9 could be regulated by distinct miRNAs depending on the cell context. Further investigation confirmed that miR-5694, but not miR-449a, targeted *AF9* mRNA in BLBC cells with a higher probability due to the consistently higher expression of miR-5694 in BLBC cells. Additionally, in clinical samples, a high *AF9* mRNA level was found to be a potential predictor of favorable DFS in overall breast cancer, and miR-5694 OE was a potential high-risk factor for a poor prognosis in BLBC patients. Notably, the *AF9* mRNA and miR-5694 expression levels correlated inversely, indicating that the destruction of the miR-5694/AF9 axis in BLBC cells could restore AF9 expression and offering a strategy for overcoming BLBC metastasis.

## Materials and Methods

The cell lines were obtained from the SIBCB (Institute of Biochemistry and Cell Biology, SIBS, CAS) cell collection or American Type Culture Collection (ATCC catalog numbers: MCF-7, T47D, BT474, MDA-MB-231, MDA-MB-468, Hs578T, and HEK293T cells). The cells were authenticated using the short tandem repeat (STR) method.

Reagents: Lipofectamine 3000 reagent was obtained from Invitrogen (Carlsbad, CA, USA).

The dual luciferase reporter assay system was purchased from Promega (Beijing, China). The radioimmunoprecipitation assay (RIPA) lysis buffer, puromycin and hygromycin were purchased from Merck/Millipore (Darmstadt, Germany).

Antibodies: The mouse monoclonal antibody against FLAG and anti-FLAG M2 affinity gel were purchased from Sigma (St. Louis, MO, USA). The rabbit polyclonal antibodies against hemagglutinin (HA) tag, CDH1 (#3195), CLDN3 (#83609), VDR (#12550), CXADR (#16984), GAPDH (#5174), CAV1 (#3238), ITGB1 (#4706), Vimentin (#5741), FN1 (#26836), and CBP (#7389) were obtained from Cell Signaling Technology (Danvers, MA, USA). The antibodies against AF9 (NBP2-15303) and Snail (AF3639) were obtained from Novus Biologicals (Littleton, USA); the ChIP antibodies POL II (05-623), H3 (06-755), H3K79me3 (07-449), and H3K9ac (07-352) were purchased from Millipore (Darmstadt, Germany). p300 (ab14984), GCN5 (ab217876), and TIP60 (ab23886) were purchased from Abcam (Cambridge, MA, UK). The IP and immunoblotting (IB) analyses were performed as previously described.^54^ Notably, the antibody usage was as follows: in IB, dilution of 1:1,000, in IP or coIP, 5 μg of each sample; and in IF or immunohistochemistry (IHC), a dilution of 1:100 or 1:200. The extraction of the total proteins with a modified buffer from RIPA lysis buffer was followed by IP and IB with the indicated antibodies. The protein levels were quantified through densitometry.

antigomiR (targeting miR-5694 or control) was commissioned to Guangzhou Ruibo Biological for synthesis.

### Plasmids, Lentivirus Package, and Infection

AF9 and Snail were cloned from the HEK293T and MDA-MB-231 cDNA libraries, respectively, using KOD FX Neo (TOYOBO) DNA polymerase and then subcloned into different Vectors. AF9 was subcloned into pCDH-3′SFB vectors to create FLAG-AF9 or subcloned into pCold-His vectors to generate His-AF9. Mitochondrial signal peptide was deleted to create the His- AF9 construct. Snail was subcloned into pWPI-5′HA to create the HA-Snail construct. The mutagenesis of FLAG-AF9 and HA-Snail was performed using a QuickChange site-directed mutagenesis kit (Stratagene, La Jolla, CA, USA). As described for the shRNA resistant clones, PCR products containing 4 synonymous mutations in the middle of the shRNA-targeted sequence were generated and subcloned into no-tagged pCDH-hygromycin vectors. The pGIPZ control was generated with the control oligonucleotide 5′-CTCGCTTGGGCGAGAGTAA-3′. pGIPZ-*AF9*-shRNA was generated with 5′-ACTTGCTCATGTCTGTTCA-3′ (shAF9#1) or 5′-GCACAG TAACATACAGCACTT-3′ (shAF9#2) oligonucleotide targeting the coding region of the AF9 transcript.

Amplification of the lentivirus was performed using standard methods in sub confluent HEK293T cells. The infection of the breast cancer cell lines was performed in the presence of polybrene (Sigma) at a final concentration of 8 μg/mL. The cells were incubated with the lentivirus mixture for 72 h and digested with trypsin to fresh growth medium; then, the cells were sorted with green fluorescence to detect stable expression or knockdown. The constructed stable cell lines were amplified and saved for future experiments.

AF9 3′ UTR and miRNAs were cloned from the genomics of MDA-MB-231 cells into the vector pCDNA3.0 for transient transfection.

### siRNA Screening with Wound Healing Assay

In total, 591 human ERF-coding genes were selected from ChromoHub.^30^ All siRNAs targeting these genes were obtained from Dharmacon (Thermo). The siRNA screening of cell migration-related genes was performed as previously described.^55^ Briefly, MCF-7 cells were plated at 12 × 10^3^ cells per well in black-walled 96-well plates (Nunc) in antibiotic-free growth medium (HyClone) 12 h before the transfection. The transfections were performed robotically using a Handler workstation SX15 with siRNAs (final concentration 25 nM) and Lipofectamine RNAiMAX (Thermo) transfection reagent (0.25 μL per well) diluted in Opti-MEM (Thermo).

When cell confluence reached 90% after the transfection, the cells were wounded by generating a longitudinal scratch under 5% variation using a robotically driven (Seiko) stainless-steel pin programmed to deliver a scratch of 0.75 × 4 mm. After wounding, the cells were washed once with growth medium and further incubated for 16 h. During this period, the real-time gap distances were imaged and determined using an IncuCyte high-throughput screening system.

### Transfection

Cells were seeded in 60 mm or 100 mm plates and transfected the following day using lipofectamine 3000 or RNAiMax (Thermo Fisher, USA) according to the instructions. A DNA (μg) to lipofectamine (μL) ratio of 1:3 was employed. For the siRNA and antigomiR transfection, a siRNA or antigomiR (nM) to RNAiMax (μL) ratio of 10:1 was employed.

### Transwell Invasion and Migration Assay

The Transwell assays of the indicated cells were performed using FalconTM Cell Culture Insert with 8 μm pores (Falcon) in serum free media according to the manufacturer’s instructions. After 24 to 48 h of incubation, the remaining cells in the upper chamber were removed with cotton swabs. The cells on the lower surface of the membrane were fixed in 4% paraformaldehyde and stained with 0.5% crystal violet. The relative cell migration was calculated by the number of migratory cells normalized to the total number of cells. In each experiment, the number of cells in five random fields (magnification, ×100) on the underside of the filter was counted. Cells in at least 3 random microscopic fields (magnification, ×10) were counted and photographed. All experiments were performed in duplicates and repeated 3 times.

### Cell Proliferation Assay

In total, 2 × 10^4^ cells were plated and counted 48 h after seeding in culture medium with 0.5% bovine calf serum. Cell proliferation was measured using Cell Counting Kit-8 (CCK-8, Dojindo Laboratories) according to the manufacturer’s instructions. The cells were plated at a density of 10^4^ cells/well in a volume of 180 μL with triplicates. On the following day, 20 μL of the CCK-8 cell-counting solution were added to each well and incubated at 37°C for 3 h. The absorbance of the solution was read spectrophoto metrically at 450 nm with a reference at 650 nm using a microtiter plate reader (Becton Dickinson).

### IF Staining

The cells were fixed and incubated with primary antibodies, Alexa Fluor dye-conjugated secondary antibodies and DAPI according to standard protocols. The cell imaging was performed under a Leica TCS SP8 WLL confocal laser scanning microscope (Leica).

### Extravasation Assays

In total, 2 × 10^6^ (in 100 μL of PBS per mouse) AF9-depleted MCF-7 cells with or without reconstituted expression of rAF9 WT or Vehicle were injected into randomized 6-week-old female athymic nude mice per group via the lateral tail veins. The mice were sacrificed after 3 h for the quantification of the total tumor cells (including intravascular and extravascular tumor cells) in lung tissues or 48 h for the quantification of extravascular tumor cells in lung tissues. The lungs were fixed in 4% formaldehyde and embedded in optimum cutting temperature (OCT) compound (Thermo) after dehydration by 30% sucrose solution. The lung tissues were systematically sectioned through the entire lung with one 50 μm section obtained in every 0.2 mm of lung thickness. The tissue sections were washed with PBS and 0.3% Triton X-100 and blocked in PBS with 10% normal goat serum, followed by incubation with the primary antibody. After washing, the samples were incubated with corresponding secondary antibodies and DAPI. Confocal images were obtained using a Leica SP8 WLL confocal laser scanning microscope (Leica, Germany) and ImageJ software.^56^^,^^57^

### Mouse Model of Tail Vein Injection-Induced Lung Metastasis and *In Situ* Intraductal-Transplantation

5- to 7-week-old female BALB/c-nu/nu nude mice were purchased from the Shanghai Institute of Material, Chinese Academy of Sciences (Shanghai, China). The mice were used in accordance with ethical regulations, and the protocol was approved by the Institutional Review Board of the Institute of Health Sciences.

For the lung metastasis, the tumors were initiated by injection of 2 × 10^6^ MCF-7 or MDA-MB-231 cells into nude mice via the tail vein. The nude mice in the control group were given 0.1 mL RPMI1640. The metastasis tumors were visualized by bioluminescence imaging of luciferase activity. Real time images are presented. Then, the tumors were dissected and snap-frozen for the molecular biology analyses. Metastasis was identified in the lung, and the numbers of metastatic tumor nodules were counted.

For the *in situ* tumor formation, the tumors were initiated by injection of 5 × 10^4^ MCF-7 or 5 × 10^3^ MDA-MB-231 cells into nude mice via intraductal-transplantation into pad. The nude mice in the control group were given 0.1 mL RPMI1640. The *in situ* tumors were removed by surgery once they reached 5 mm^2^. After 75 days or 25 days with the MCF-7 or MDA-MB-231 cells, respectively, the metastatic tumors were first visualized by bioluminescence imaging of luciferase activity. Then, the tumors were dissected and snap-frozen for the molecular biology analyses. The bone metastatic site was identified by H&E staining, and the relative metastatic area was counted and presented as a ratio of the whole hind leg bone.

### Bioluminescence Imaging with IVIS

Mice were intraperitoneally injected with 100 μL of 7.5 mg/mL D-luciferin (Xenogen) and subsequently anesthetized with isoflurane inhalation. Bioluminescence imaging with a CCD camera (IVIS, Xenogen) was initiated 10 mins after the injection. Bioluminescence from the region of interest was defined manually. The background was defined using a region of interest from a mouse that was not given an intraperitoneal injection of D-luciferin. All bioluminescent data were collected and analyzed using IVIS-Image software.

### IP and LC-MS/MS

FLAG mouse mono-antibody coated beads were incubated with FLAG-AF9 cell lysate for 4 hr. Purified FLAG-AF9 and its associated complexes were boiled at 95°C for 8 min and then loaded onto SDS-PAGE gel for the separation of FLAG-AF9 and its associated proteins. This SDS-PAGE gel sample was processed through a series of routine flow, such as reductive alkylation, trypsin digestion, and peptide extraction. The peptides were analyzed by LC-MS/MS using a Q Exactive mass spectrometer (Thermo Fisher Scientific, Waltham, MA). The proteins were identified by a database search of the fragment spectra against the National SwissProt protein database (EBI) using Mascot Server 2.4 (Matrix Science, London, UK).

### TF Enrichment of AF9 Target Genes

TRRUST (Transcriptional Regulatory Relationships Unraveled by Sentence-based Text mining) v2 database (www.grnpedia.org/trrust) is a manually curated database used to analyze potential TFs regulating AF9 target genes. The TRRUST v2 database, which utilizes a sentence-based text mining approach, includes 8444 TF-target interactions of 800 human TFs.^58^ With its friendly web interface, we input a list of AF9 target genes and acquired the TF-target interaction results ranked by the q value (q values less than 0.0001 were considered statistically significant).

### Dual-Luciferase Reporter Assay

For the dual luciferase reporter assay in each group, the pGL3-derived reporter construct pGL3-*CDH1* promoter (−57 to +149 relative to TSS) was cotransfected into MDA-231, Hs578T, or MDA-157 cells in 24-well plates with Renilla luciferase plasmid pRL-TK. After the transfection with the indicated plasmids for 48 h, the cells were washed with cold PBS and lysed by passive lysis buffer. Luciferase activity was measured using a dual-luciferase assay kit (Promega) on a GloMax 20/20 luminometer (Promega) following the manufacturer’s instructions. The relative levels of luciferase activity were normalized to the levels in the untreated cells and levels of luciferase activity in the Renilla control plasmid in each group.

### RNA Sequencing

The total RNA was extracted for the RNA sequencing on an Illumina HiSeq 2500 system. The sequencing data analysis and management were performed with BaseSpace Sequence Hub. A KEGG pathway analysis was performed using the software clusterProfiler,^59^ and the plot was generated using ggplot2 in R (3.5.0). A GSEA was conducted using the software GSEA (v4.1.0) as described in a previous report.^60^

### Quantitative Reverse Transcriptase PCR Analysis of mRNA and miRNA

The total RNA was extracted with an RNA High-Purity Total RNA Rapid Extraction Kit (QIAGEN). cDNA was prepared by using oligonucleotide (dT), random primers, and a Thermo Reverse Transcription kit (Roche). The quantitative real-time PCR analysis was performed using 2× SYBR real-time PCR Premixture (Roche) under the following conditions using an ABI Prism 7700 sequence detection system: 5 min at 95°C, followed by 40 cycles at 95°C for 30 s, 55°C for 40 s, and 72°C for 1 min. The quantitation of the miRNAs by qRT-PCR was performed according to a previous report.^61^ The data were normalized to the expression of a control gene (GAPDH) in each experiment. The data represent the mean ± SD of three independent experiments. The primer pairs used for quantitative real-time PCR are listed in Table S1.

### miRNA Target Prediction

We used the miRDB database (http://mirdb.org/index.html) to predict the potential miRNAs regulating AF9 gene expression. The miRDB database, which includes support vector machines (SVMs) and high-throughput training data, can calculate thousands of miRNA-target interactions.^62^^,^^63^ Based on this method, we acquired a list of miRNAs that potentially target AF9 mRNA, and we retained those miRNAs with prediction scores greater than 80 (strong binding).

Furthermore, given that AF9 expression is significantly downregulated in breast cancer, we performed a differential expression analysis (DEA) of all miRNAs within the TCGA-BRCA cohort and acquired a list of miRNAs that were significantly upregulated in breast cancer (p < 0.001). Finally, we integrated the predicted miRDB miRNA data with the TCGA-BRCA DEA results and identified two miRNAs (hsa-mir-5694 and hsa-mir-449a) that strongly bind the AF9 mRNA 3′ UTR and are significantly upregulated in breast cancer.

### ChIP Assay

The ChIP assay was performed according to routine operation. Rabbit monoclonal anti-Snail (1:500; Cell Signaling), anti-FLAG (1:2000, Sigma), rabbit monoclonal anti-H3K9ac (1:500; Millipore), and rabbit monoclonal anti-H3K79me3 (1:500; Millipore) antibodies were used in the ChIP assays with a rabbit monoclonal immunoglobulin G (IgG) (1:500; Cell Signaling) as a negative control. The presence of binding regions detected by the indicated antibodies was assessed by PCR. A small amount of precleared DNA (before the addition of the antibodies) was set as an input control and used to normalize the ChIP enriched DNA. The PCR primer sequences of the DNA fragments as parts of the targeted promoters are provided in Table S1. The ChIP signal from the control groups was set to 1.00. The other groups were relative to the corresponding controls.

### Clinical and Transcriptomic Characteristics of TCGA Breast Cancer Cohort

TCGA Breast Cancer (BRCA) datasets were collected from the UCSC Xena multiomics database platform (https://tcga.xenahubs.net),^64^ which included patients’ clinical annotated phenotypes (n = 1,247) and corresponding RNA sequencing transcriptomic features (n = 1,218). The BRCA expression profile in this database platform also included both gene-level expression data (gene expression RNA-seq-IlluminaHiSeq, n = 1,218) and miRNA-level transcription estimates (miRNA mature strand expression RNA-seq-IlluminaHiseq, n = 832), which were previously preprocessed, annotated, and normalized.^64^ Therefore, based on the matched TCGA sample IDs of these data, we could stratify these BRCA samples into two groups (primary tumors versus adjacent normal tissues based on the “sample type” phenotype) and four groups (BRCA subtypes and pathologic stages based on the “PAM50_mRNA_nature2012,”^42^ “AJCC_Stage_nature2012,”^42^ and “Tumor_nature2012”^42^ phenotypes) and then compare the transcriptomic expression values. Because there were no miRNA expression values for the BRCA “Normal-like” subtype, we did not perform an MLLT3-expression analysis of this subtype. Furthermore, we did not analyze tumors with undetermined pathologic stages, such as “Stage X” and “TX.”

### Collection of Clinical Breast Cancer Samples and IHC Analysis

This study was approved by the Institutional Review Board of The First Affiliated Hospital of China Medical University. Each patient provided signed informed consent. The diagnoses of the breast cancer samples were verified by pathologists. All patients received standard chemotherapy after surgery. The use of the tissue materials for research was approved by the Ethics Committee of The First Affiliated Hospital of China Medical University. Breast cancer is staged on a scale from zero to four. Stages 0 to III are defined as nonmetastatic. In Stage IV, the cancer has spread to other organs of the body, most often the bones, lungs, or liver; thus, a cancer sample in stage IV is defined as metastatic.

Tissue sections from paraffin-embedded human breast cancer patient specimens were stained with antibodies as indicated. We quantitatively scored the tissue sections according to the percentage of positive cells and staining intensity. We rated the intensity of the staining on a scale from 0 to 3 as follows: 0, negative; 1, weak; 2, moderate; and 3, strong. We assigned the following proportion scores: X indicates X% of the tumor cells were stained (0 ≤ X ≤ 100). The score (H-score) was obtained by the following formula: 3 × percentage of strongly staining nuclei + 2 × percentage of moderately staining nuclei + 1 × percentage of weakly staining nuclei, yielding a range from 0 to 300. The scores were compared with OS, which was defined as the time from the date of diagnosis to death or last known date of follow-up. We compared the survival durations of 138 patients, all of whom received appropriate therapy, with low (0–150 staining) versus high (150.1–300 staining) AF9 expression.

### Statistical Analysis

The statistical analysis was performed using Statistical Package for the Social Sciences (SPSS) software version 12 for Windows (SPSS, Chicago, IL, USA). Student’s t tests were used to determine the statistical significance of the differences between the experimental groups. A p value <0.05 was considered significant. Graphs were created using Microcal Origin software (version 3.78; Microcal Software, Northampton, MA, USA). We used Kaplan-Meier Plotter (https://kmplot.com/analysis/index.php?p=service&cancer=breast),^65^ which is an online survival analysis tool, to assess the association between MLLT3/AF9 expression (the Affy ID: 204917_s_at) and OS (split patients by: median; follow up threshold: 180 months).

## Supplemental Information

Document S1. Figures S1–S7 and Table S1

Document S2. Article plus Supplemental Information
