# Supplementary material for: The miR-5694/AF9/Snail Axis Provides Metastatic Advantages and a Therapeutic Target in Basal-like Breast Cancer
Source: Mol Ther. 2020 Nov 20;29(3):1239–57. doi: 10.1016/j.ymthe.2020.11.022 (PMC7934584; doi:10.1016/j.ymthe.2020.11.022)
Supplement: Document S1. Figures S1–S7 and Table S1 [file mmc1.pdf]

YMTHE, Volume *29*

Supplemental Information

The miR-5694/AF9/Snail Axis Provides Metastatic Advantages and a Therapeutic Target in Basal-like Breast Cancer

Xin Tian, Hua Yu, Dong Li, Guojiang Jin, Shundong Dai, Pengchao Gong, Cuicui Kong, and Xiongjun Wang

Supplementary Figure and Figure Legends

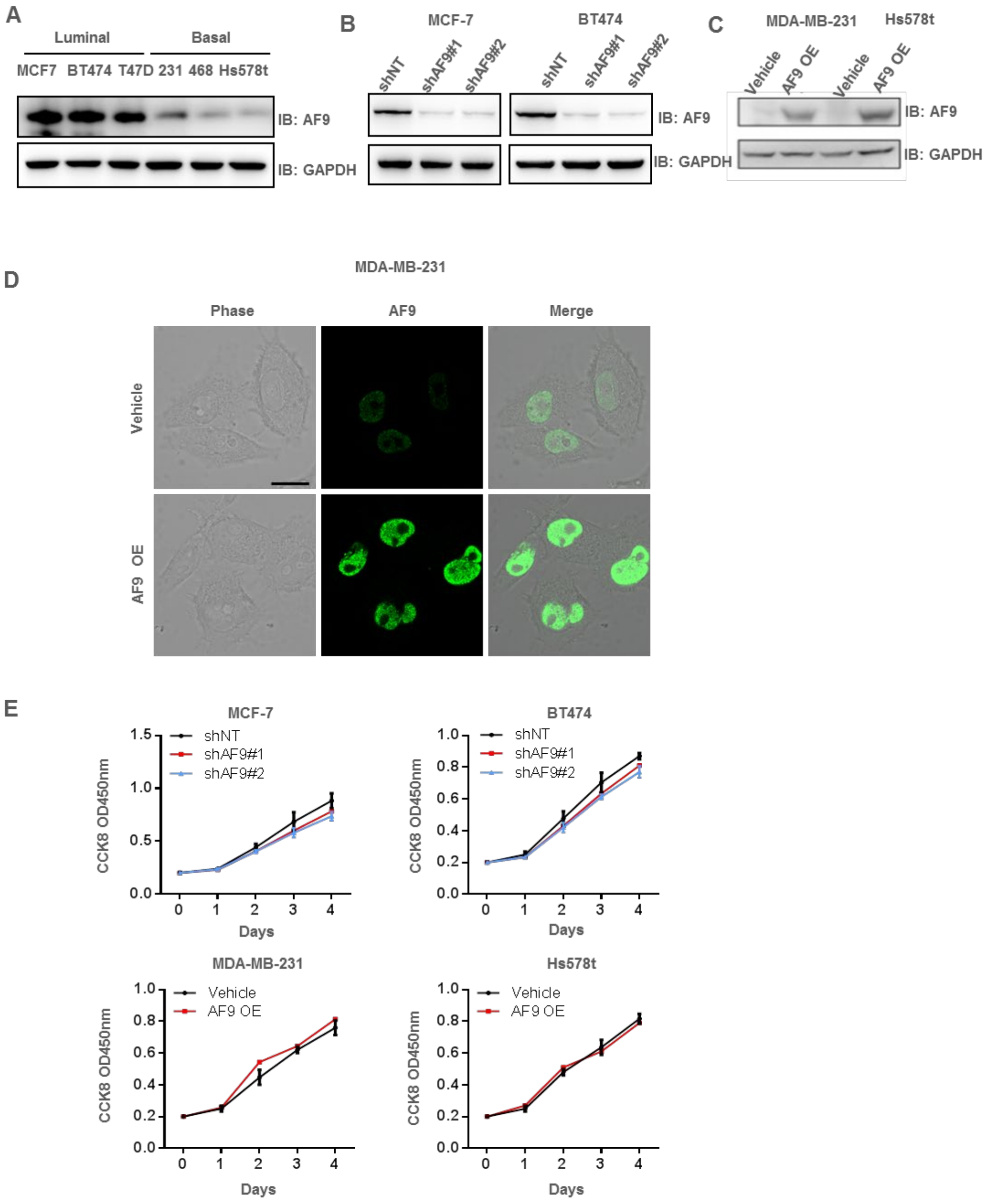

Fig S1. Referred to Figure 1.

(A-C), Western blot was performed with the indicated antibodies.

(A), AF9 protein levels in three luminal breast cancer cell lines and three basal like breast cancer cell lines.

(B), Knocking down of AF9 in MCF-7 and BT474 cell lines.

(C), Forced expression of AF9 in MDA-MB-231 and Hs578t cell lines.

(D), Representative images of IF staining performed in MDA-MB-231 Vehicle or AF9 OE cells with indicated antibodies. Scale bar, 20 μm.

(E), Cell proliferation assays were performed using MCF-7-shNT cells and MCF-7-shAF9#1,

#2 cells, or T47D-shNT cells and T47D-shAF9#1, #2 cells, or MDA-MB-231-Vehicle and MDA-MB-231-AF9 OE, or Hs578T-Vehicle and Hs578T-AF9 OE cells. Error bars represent standard deviation (SD).

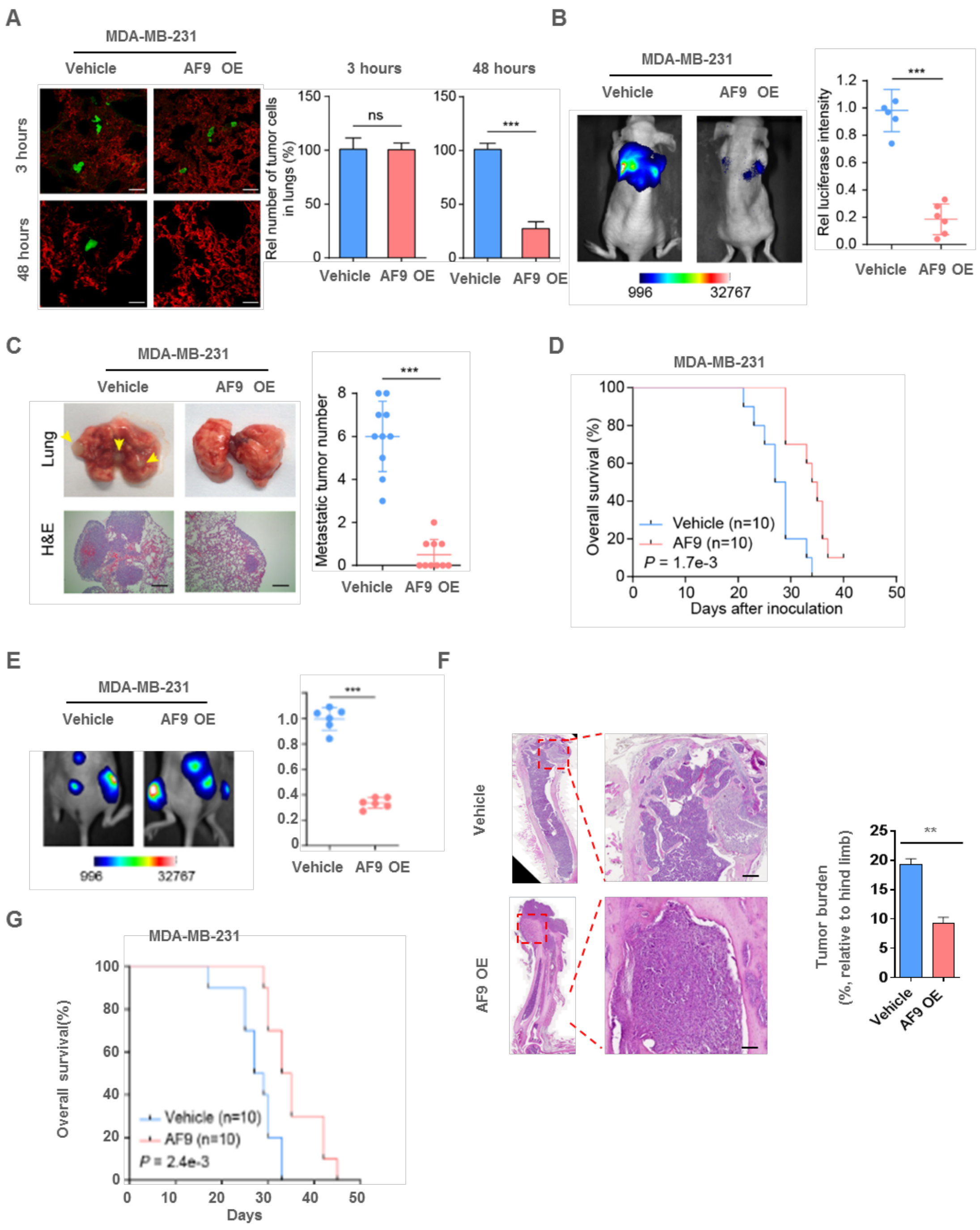

Figure S2. Forced expression of AF9 impaired metastatic capacity of BLBC cells *in vivo*.

(A), Extravasation analysis of MDA-MB-231 cells. MDA-MB-231-Vehicle cells or

MDA-MB-231-AF9 OE cells were implanted into randomized athymic nude mice via tail vein injection (5 mice per group). 3 or 48 hrs after inoculation, the mice were sacrificed. Representative images of extravascular tumor cells (green) out of blood vessels (red) were shown (A, left panel). The numbers of extravascular tumor cells were quantified (A, right panel). Scale bar, 20 μm.

(B-C), Mouse model of lung metastasis. MDA-MB-231-Vehicle cells or MDA-MB-231-AF9 OE cells were implanted into nude mice via tail vein injection (6 mice per group). 25 days after inoculation, bioluminescence imaging of these implanted mice was carried out and representative images of lung metastasis were presented (B, left panel). The luciferase intensities of metastatic tumors in lungs were statistically analyzed (B, right panel). Representative images of lung tissues dissected 40 days after inoculation and H&E stained metastatic nodules were presented (C, left panel) and calculated by counting the surface tumors in lungs(C, right panel). Data represent the mean ± SD of the luciferase intensities in 6 mice. Scale bar, 200 μm.

(D), Survival durations. Kaplan-Meier survival analysis of another batch of mice (10 mice per group) implanted with the following tumor cells as described in Figure. S2A.

(E-F), Mouse model of *in situ* intraductal-transplantation. Tumors were initiated by injection of 5×10^3 MDA-MB-231 cells into nude mice via intraductal-transplantation. Nude Mice in the control group were given 0.1 ml L-15 Medium. The in situ tumors were dissected and removed by surgery when reaching 5 square millimeters. After 25 days, the metastatic tumors were first visualized by bioluminescence imaging of luciferase activity. Real time images were presented and intensities of images were shown (E). Then, tumors were dissected and snap-frozen for molecular biology analyses. The bone metastatic site was identified by H&E and the relative metastatic area was counted and presented a ratio versus the whole hind leg bone (F). Scale bar, 200 μm.

(G), Survival durations. Kaplan-Meier survival analysis of another batch of mice (10 mice per group) implanted with the following tumor cells as described in Figure. S2A.

A, B, C, E, and F, Two tailed Student's t-test. Error bars represent standard deviation (SD).

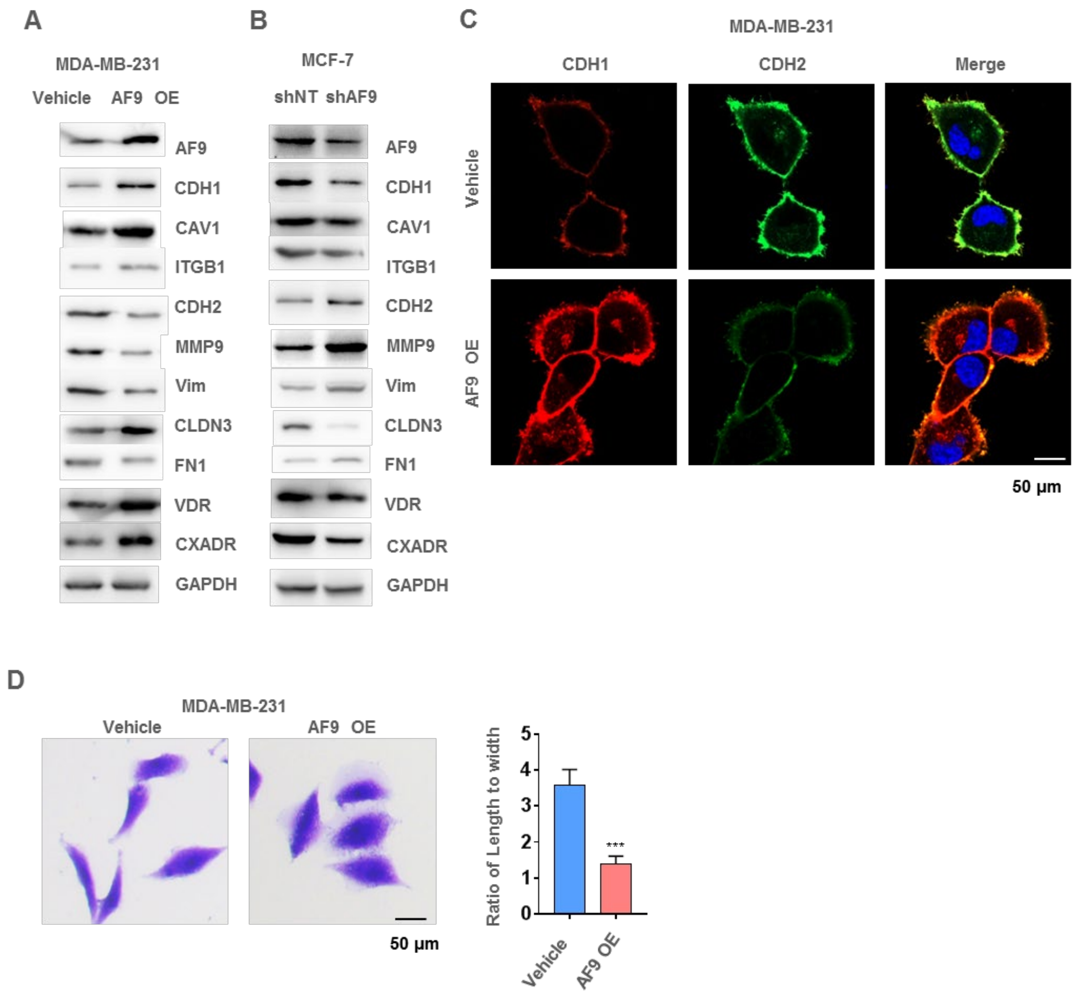

Fig S3. AF9 expression level determines the migration ability of MDA-MB-231 and MCF7 cells.

(A-B), Western blot was performed with the indicated antibodies.

(A) and (B), Western blot was performed using indicated protein in MDA-MB-231 cells Vehicle and AF9 OE cells or in MCF-7 shNT and shAF9 cells with or without knocking down AF9.

(C), Representative images of IF staining performed in MDA-MB-231 Vehicle or AF9 OE cells with indicated antibodies. Scale bar, 20 μm.

(D), Cellular morphology was recorded and ratio of width to length of cells was analyzed in indicated MBA-MD-231 cells. Cells were visualized by crystal violet staining. Data are means ± SEM (n = 50). **P< 0.001. Scale bar, 50 μm. Two tailed Student's t-test. Error bars represent standard deviation (SD).

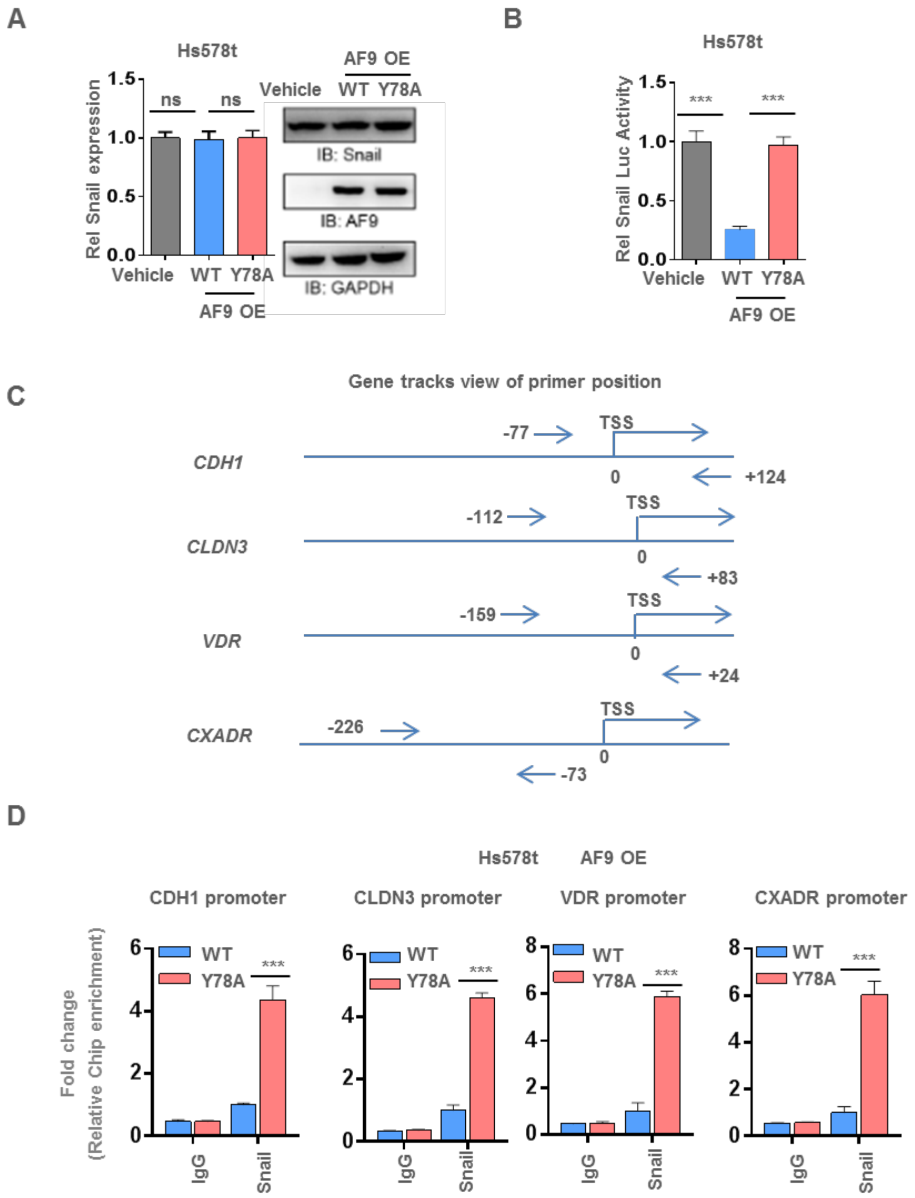

Figure S4. Referred to Figure 4.

(A), Snail mRNA expression and protein level were tested in Hs578t-Vehicle and Hs578t-AF9 OE (WT or Y78A) cells.

(B), Snail transcriptional activity was determined in Hs578t-Vehicle and Hs578t-AF9 OE (WT or Y78A) cells by dual luciferase assay. These cells were co-transfected with Snail-Luc plasmid (pGL3-*CDH1* promoter (-57 to +149)) and Renilla control plasmid pRL-TK for 48 hours. Data represent the mean ± SD of three independent experiments.

(C), Gene tracks view of primers against promoter regions of *CDH1*, *CLDN3*, *VDR* and *CXADR*.

(D), Ch-IP assay was performed in Hs578t-AF9 OE (WT or Y78A) cells with indicated

antibodies. Primers against promoter regions of *CDH1*, *CLDN3*, *VDR* and *CXADR* were used to perform quantitative real-time PCR to measure the binding affinity of Snail. The relative binding affinities of Snail to target region were normalized to IgG.

A, B, and D, Two tailed Student's t-test. Error bars represent standard deviation (SD).

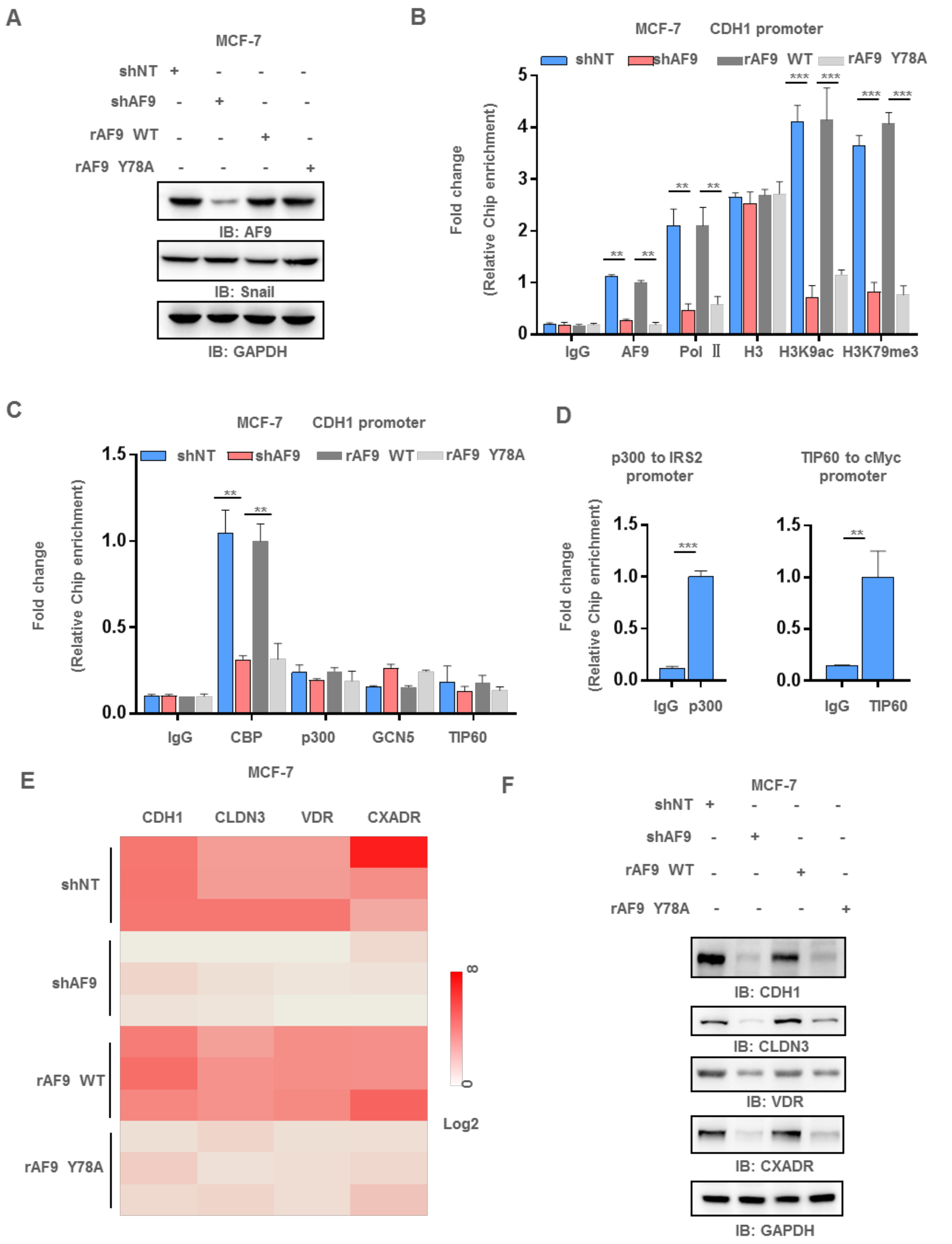

Figure S5. AF9 is required for luminal breast cancer cells expressing *CDH1, CLDN3, VDR* and *CXADR*.

(A), Restored expression of AF9 (including WT or Y78A mutant) in shAF9 cells.

(B) and (C), Ch-IP assay was performed in MCF7-shNT, MCF7-shAF9 or MCF7-shAF9+ (WT or Y78A) cells with indicated antibodies. Quantitative real-time PCR using primers against promoter regions of *CDH1* measured the binding affinity of H3K9ac and H3K79me3, and histone acetyltransferases: CBP, P300, GCN5 and TIP60 on the *CDH1* promoter. Antibodies against Pol Ⅱ and H3 were used as inner control for gene expression and histone association with the *CDH1* promoter. The relative binding affinities were normalized to IgG and then to 5% input.

(D), The positive controls for Ch-IP assay of p300 and TIP60 by targeting the promoters of IRS2 and cMyc, respectively.

(E) and (F), The mRNA and protein levels of CDH1, CLDN3, VDR and CXADR were tested in MCF7 cells with indicated manipulations.

B, C, and D, Two tailed Student's t-test. Error bars represent standard deviation (SD).

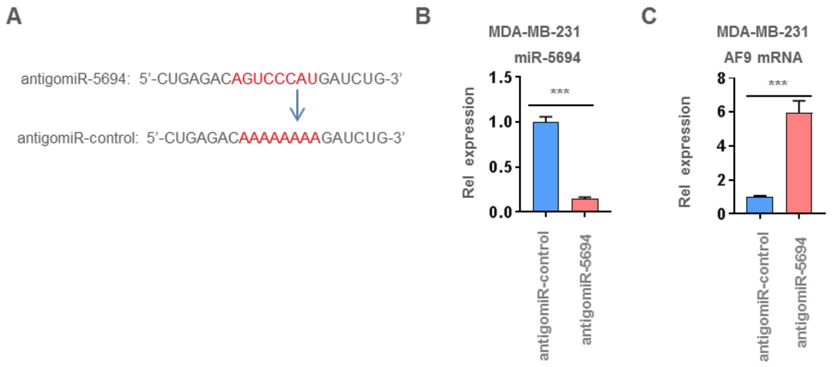

Figure S6. AntigomiR against miR-5694 de-repressed AF9 expression in MDA-MB-231 cells.

(A), Schematic diagram of synthetic antigomiR against miR-5694(named as antimiR-5694) and the indicated control (named as antigomiR-control).

(B) and (C), qPCR assay for testing miR-5694 or AF9 mRNA was performed in cells transfected with antimiR-5694 or antigomiR-control.

B and C, Two tailed Student's t-test. Error bars represent standard deviation (SD).

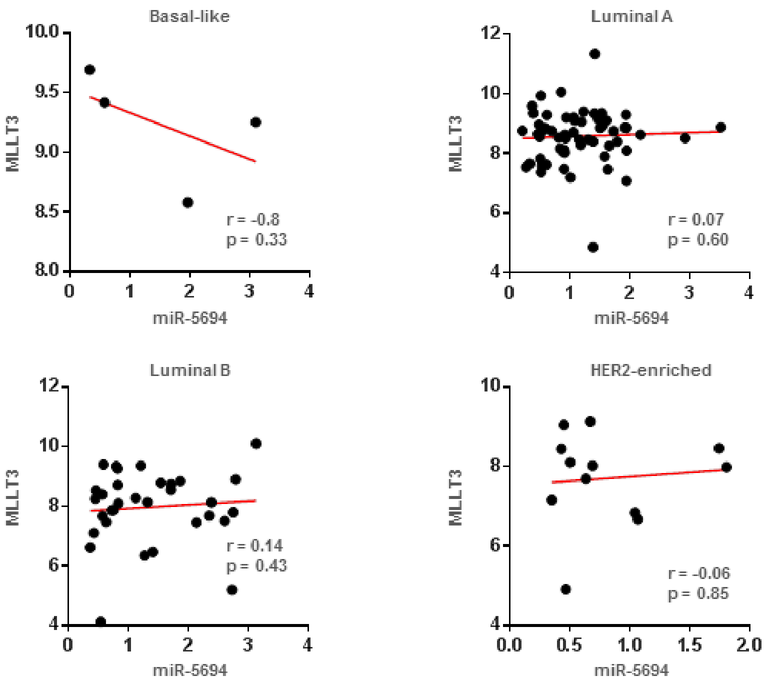

Figure S7. Referred to Figure 7. Scatter plots of MLLT3 and miR-5694 expression among four breast cancer subtypes within the TCGA-BRCA cohort. The red line indicates the fit of linear regression; r is the Spearman correlation coefficient.

Supplementary Table 1

qPCR primers for testing gene expression:

AF9-F 5'- TTTGTGGAGAAAGTCGTCTTCC
AF9-R 5'- GAGGTGATTCACTGGTGGATG

CDH1-F 5'-ATTCTGATTCTGCTGCTCTTG
CDH1-R 5'-AGTAGTCATAGTCCTGGTCTT

SNAIl-F 5'- TCGGAAGCCTAACTACAGCGA
SNAIl-R 5'- AGATGAGCATTGGCAGCGAG

CDH2-F 5'-TCAGGCGTCTGTAGAGGCTT
CDH2-R 5'-ATGCACATCCTTCGATAAGACTG

CAV1-F 5'-CATGCCTGTCATACCACAAC
CAV1-R 5'-GGTGTCAGATGGAGGAGGG

ITGB1-F 5'- CCTACTTCTGCACGATGTGATG
ITGB1-R 5'- CCTTTGCTACGGTTGGTTACATT

MMP2-F 5'- TTCCCAAATTATGTCTCCCTGGA
MMP2-R 5'- ATGGGGTATAGTGGGTTCCTTT

MMP9-F 5'-T GTACCGCTATGGTTACACTCG
MMP9-R 5'- GGCAGGGACAGTTGCTTCT

VIM-F 5'-ATTGCCACCTACAGGAAGCT
VIM-R 5'-GCAGAAAGGCACTTGAAAGC

PTK2-F 5'- TGGTGCAATGGAGCGAGTATT
PTK2-R 5'- CAGTGAACCTCCTCTGACCG

CLDN1-F 5'- GAGGCTCCGATAAAGCCAAAG
CLDN1-R 5'- ACAGAGCGGCTCCTAATTCAT

CLDN3-F 5'- AACACCATTATCCGGGACTTCT
CLDN3-R 5'- GCGGAGTAGACGACCTTGG

ACTN4-F 5'- GCAGCATGGGCGACTACAT
ACTN4-R 5'- TTGAGCCCGTCTCGGAAGT

FN1-F 5'- AAGATAACCGTGTGATGCAGTT
FN1-R 5'- GGGGAGCAGGTAATGACGTATTT

VDR-F 5’- GTGGACATCGGCATGATGAAG
VDR-R 5’- GGTCGTAGGTCTTATGGTGGG

CXADR-F 5’- GTTTCCCCGCCTGAGCTAAC
CXADR-R 5’- TTCTGGAAGCGCCCAATAGG

TUBB-F 5’-TGGACTCTGTTCGCTCAGGT
TUBB-R 5’TGCCTCCTTCCGTACCACAT

qPCR primers for testing miR-449a:
miR-449a-F 5’-GCAGATCATGGGACTGTC
miR-449a-R 5’-GTCCAGTTTTTTTTTTTTTTTCTGAG

qPCR primers for testing miR-5694:
miR-5694-F 5’-GCAGTGGCAGTGTATTGTTAG
miR-5694-R 5’-GTCCAGTTTTTTTTTTTTTTTACCAG

qPCR primers for testing Ch-IP assay:
Primers for amplifying DNA product in the *CDH1* promoter
F (-77): 5’-GTGAACCCTCAGCCAATCAGC
R (+124):5’-GGCTGGCCGGGGACGCCGAGC

Primers for amplifying DNA product in the *CLDN3* promoter
F (-112): 5’-AGGCAGGGGCCACGTCACTGT
R (+83): 5’-TAACGGCTCGGCTCCATACGC

Primers for amplifying DNA product in the *VDR* promoter
F (-159): 5’-GATGGTTGCAGCGCCAACGGA
R (+24): 5’-GTGGACAAGCTGTTCCGCGCT

Primers for amplifying DNA product in the *CXADR* promoter
F (-226): 5’-CCCGCGACCTACGACGCCGCG
R (-73): 5’- TCACTTCCGGCAGCGGCCCGG
